# Diverse effects of degree of urbanisation and forest size on species richness and functional diversity of plants, and ground surface-active ants and spiders

**DOI:** 10.1371/journal.pone.0199245

**Published:** 2018-06-19

**Authors:** Ramona Laila Melliger, Brigitte Braschler, Hans-Peter Rusterholz, Bruno Baur

**Affiliations:** Section of Conservation Biology, Department of Environmental Sciences, University of Basel, Basel, Switzerland; Charles University, CZECH REPUBLIC

## Abstract

Urbanisation is increasing worldwide and is regarded a major driver of environmental change altering local species assemblages in urban green areas. Forests are one of the most frequent habitat types in urban landscapes harbouring many native species and providing important ecosystem services. By using a multi-taxa approach covering a range of trophic ranks, we examined the influence of degree of urbanisation and forest size on the species richness and functional diversity of plants, and ground surface-active ants and spiders. We conducted field surveys in twenty-six forests in the urban region of Basel, Switzerland. We found that a species’ response to urbanisation varied depending on trophic rank, habitat specificity and the diversity indices used. In plants, species richness decreased with degree of urbanisation, whereas that of both arthropod groups was not affected. However, ants and spiders at higher trophic rank showed greater shifts in species composition with increasing degree of urbanisation, and the percentage of forest specialists in both arthropod groups increased with forest size. Local abiotic site characteristics were also crucial for plant species diversity and species composition, while the structural diversity of both leaf litter and vegetation was important for the diversity of ants and spiders. Our results highlight that even small urban forests can harbour a considerable biodiversity including habitat specialists. Nonetheless, urbanisation directly and indirectly caused major shifts in species composition. Therefore, special consideration needs to be given to vulnerable species, including those with special habitat requirements. Locally adapted management practices could be a step forward to enhance habitat quality in a way to maximize diversity of forest species and thus ensure forest ecosystem functioning; albeit large-scale factors also remain important.

## Introduction

Urbanisation is increasing globally and is considered a main driver of environmental change [1]. Urbanisation-related factors including reduced habitat size and increased spatial isolation change the dynamics of plant and animal populations in urban green areas [2, 3]. Several studies along urbanisation gradients also reported alterations in abiotic conditions in the remaining habitat patches caused by increases in temperature, precipitation and N deposition from the rural surroundings to the city centre [1, 4, 5]. These changes influence habitat quality and, consequently, the species richness, species composition and functional diversity of plants and animals [3, 6, 7], which in turn affect the functioning of ecosystems [8]. Furthermore, urbanisation can influence the population dynamics of animals and plants by altering the biology of hosts, pathogens and vectors [9]. Although urbanisation frequently reduces the abundance of many parasites and pathogens [10], transmission may also increase among urban-adapted hosts [9]. In some cases, invertebrates can serve as vectors of pathogens which otherwise are absent from urban environments [11]. Finally, plants and animals may be exposed to other chemicals (herbicides, fungicides, pesticides) and other types of pollution in urban environments than in rural agricultural landscapes [9]. Nonetheless, urban areas can harbour remarkably high species richness [12], in some cases exceeding that of their rural surroundings [6, 13].

Forests represent one of the most frequent types of green area in cities [14]. Urban forests provide a wide range of ecosystem functions including habitat for native species and recreation for residents [15, 16]. Both forests and orchards in cities can serve as refugia for rare and threatened specialist species and thus can be of high conservation value [12, 17]. Within urban landscapes, forest sites differ substantially in site history, management and disturbance intensity and consequently in species composition [3, 4, 17, 18]. Urban forests can be remnants of former continuous forests, a result of ongoing succession or actively planted [4]. They can include urban orchards, cemeteries overgrown by trees, or parks [4, 17, 18].

Not all species respond to environmental changes caused by urbanisation in the same way, because they have different requirements regarding their habitat and its surrounding landscape [7, 12, 19]. For example, specialist species may perceive the surrounding matrix as a stronger barrier than generalists, which are able to exploit a wide variety of resources from neighbouring green areas [1, 16, 20]. Thus, specialist species become frequently replaced by generalists [21, 22]. As a result, species composition in urban areas becomes more and more similar, which in turn may lead to a decrease in functional diversity–also called functional homogenisation ([20] and references within). Furthermore, groups of species at high trophic ranks such as herbivores and predators might also be more influenced by increased isolation and habitat loss because of their dependence on other species compared to groups of species at low trophic ranks such as plants [23, 24].

The majority of urban forest studies focused on a single taxonomic group, frequently plants, butterflies, carabids or birds (e.g. [22, 25, 26] and reviews of [27, 28]) or higher taxon or morphospecies levels [29, 30]. So far, few studies have examined the impact of urbanisation on the species diversity and/or functional diversity in forests using a multi-taxa approach. These studies often investigated either taxonomic groups at similar trophic ranks like carabids, rove beetles and spiders [31, 32, 33] or carrion-burying beetles, their phoretic mites, and muscoid flies [34] or focused on species with mutualistic or exploitative relationships [34, 35] or with similar life-history traits [20]. Most multi-taxa urban studies were conducted in openland habitats [19, 29, 35] or over a variety of habitat types [7]. To our knowledge, no studies were conducted in different-sized urban forests and considered species groups with different trophic ranks.

In this study, we examined the impact of degree of urbanisation and forest size on the species and functional diversity of vascular plants and ground surface-active ants and spiders in forest sites in the city of Basel (Switzerland) and its suburban surroundings. The forests examined in our study are very small and embedded in a small-scattered landscape, where settlements and green areas are located within short distances. A rural–urban gradient approach extending over several kilometres is, therefore, not appropriate in our study area. Instead, we used the percentage cover of sealed area in the closer surroundings of the forests as a measure of degree of urbanisation as suggested by others (e.g. [31, 36]).

The taxonomic groups considered in our study vary in trophic rank and thus in the use of resources available in the urban landscape. Ants are intermediate between the other two groups, as many ant species not only consume animal matter but also some plant material such as nectar or elaiosomes attached to seeds. Many species indirectly consume plant sap as excretion from sucking insects. In contrast, spiders are predators. Neither of the two arthropod groups depends on specific plant species as a resource. Hence, their responses to urbanisation can be expected to be independent of that of plants.

In particular, we hypothesize that the diversity of plants, ants, and spiders (species richness, Shannon diversity and evenness and functional diversity) decrease with both increasing degree of urbanisation and decreasing forest size. These effects will be more pronounced for ants and spiders, because of their higher trophic rank, and for forest specialists due to their narrow habitat range. We further expect that small forests show lower species diversity and thus altered functional diversity and harbour lower percentages of forest specialists in highly compared to less urbanised forest areas. In contrast, the diversity in large forest sites should be less negatively affected by degree of urbanisation.

Secondly, we hypothesize that species composition of plants, ants, and spiders will be altered by the degree of urbanisation and forest size. We expect that species composition in highly urbanised areas will be more similar than in less urbanised areas.

## Methods

### Study area

**Fig 1:**
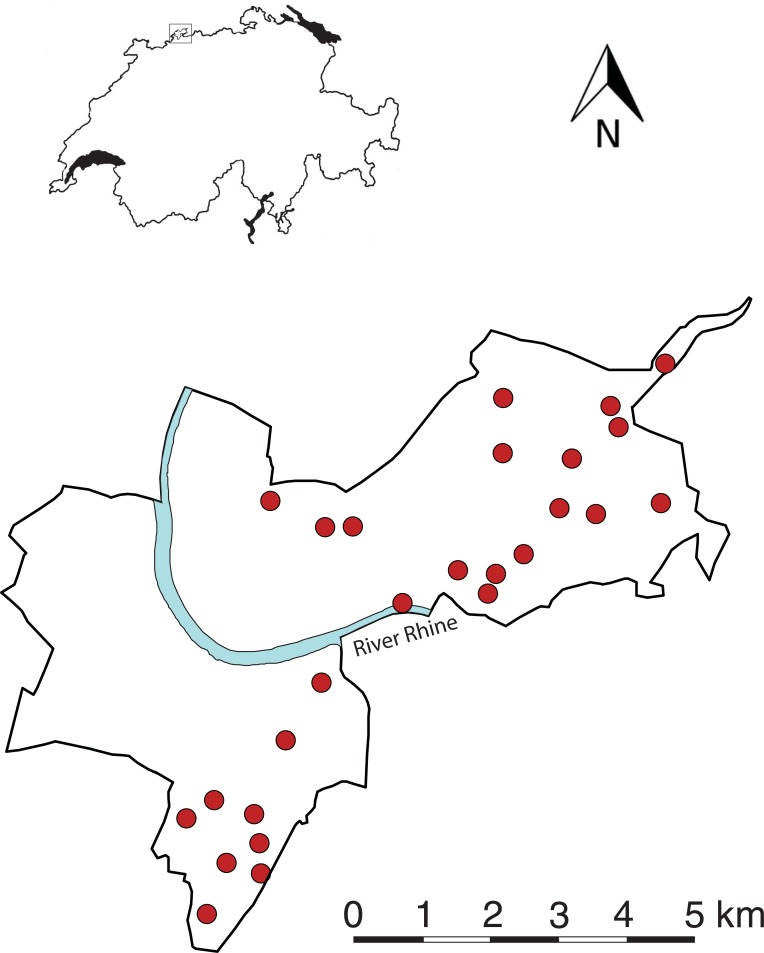
Location of the study area in Northwestern Switzerland and the distribution of the forests examined in the area of Basel-Stadt. The investigation area is surrounded by dense settlements in Germany (north), France (northwest) and Switzerland (south-west).

The study was conducted in the canton Basel-Stadt (comprising the city of Basel and the municipalities Riehen and Bettingen; hereafter referred to as Basel, Fig 1), Switzerland (47°34’N, 7°36’E, elevation: 245–522 m a.s.l.). The study area covers 37 km^2^, consisting of 26.3 km^2^ (70.9%) residential area, 4.5 km^2^ (12.1%) agricultural land, 4.4 km^2^ (11.7%) forest and 1.7 km^2^ (4.5%) water bodies (Statistisches Amt Kanton Basel-Stadt: www.statistik-bs.ch). Basel has 196,471 inhabitants and a population density of 5320 inhabitants km^-2^ (www.statistik-bs.ch). Total annual precipitation averages 842 mm and annual mean temperature is 10.5°C (records from 1981 to 2010, www.meteoswiss.admin.ch). Most study sites were state owned and accessible to the public. Some forest was privately owned but managed by the forestry authorities. Permission for fieldwork was obtained from landowners, managers, and the authority responsible for the forests (Amt für Wald beider Basel).

### Characteristics of the forests

To investigate the potential effects of degree of urbanisation and forest area on the species diversity of vascular plants, and soil surface-active ants and spiders, we chose 26 deciduous forests, belonging to the *Fagetum* association [37] and ranging in size from 258 m^2^ to 50,000 m^2^ (Fig 1; S1 Table; S2 Table). The forest sites examined differ in their historical development and consequently in age. Twenty of them are surrounded by settlements and agricultural lands and are no longer connected to large continuous forests (> 40 ha). These forest patches are either remnants of former large continuous forests (fragments) or a result of abandonment of orchards or planted after 1884 (planted; see S1 Table and S2 Table for detailed description of forests). For each of these twenty forests, we calculated the shape index following Gyenizse et al. [38]. A shape index of 1 corresponds to a circular area, which is considered as most stable and resistant against biotic and abiotic effects from the surrounding landscape [38].

### Vegetation survey

In each forest, we installed six sampling plots measuring 4 m × 4 m. Plots had a minimum distance of 1 m to the forest edge or permanent trails to minimize potential edge effects. We assessed species richness of vascular plants in the ground vegetation (≤ 40 cm) and cover of single species in a 2 m × 2 m subplot established in a randomly chosen corner of each 4 m × 4 m plot using the Braun-Blanquet scale [39]. To complete the plant species list in the entire sampling plot, additional species found in the other three 2 m × 2 m subplots were recorded.

### Ant and spider sampling

We conducted pitfall trapping to sample ground surface-active ants and spiders. We installed a trapping grid in each of the forests examined. We arranged twelve pitfall traps (plastic cups: 5.8 cm diameter; fluid: 60 ml of water–detergent solution) in two rows with six traps each in a trap-grid system. The distance of the traps between and within the rows was 5 m. The size of the pitfall grid was determined by the smallest fragment, which was thus comprehensively sampled. A dummy of a grid of corresponding size was placed with closed eyes on a map showing forest cover and paths for the larger fragments thus avoiding prior knowledge of vegetation cover or topography when selecting the location for the pitfall trap grids. If necessary the grid was moved to be entirely within the forested area. To account for seasonal differences in activity among species, we operated pitfall traps once in spring, three times consecutively in summer, and once in autumn 2014. Traps were exposed for 7 days before being collected, which resulted in a maximum of 60 trap weeks per forest site (12 traps × 5 sampling weeks).

We transferred trap contents to 70% ethanol for further processing. We identified individuals to the species level following the keys of Seifert [40] and Ward et al. ([41]; *Colobopsis truncata*) for ants and Roberts [42, 43] and Nentwig et al. ([44], <www.araneae.unibe.ch>, version 03.2017) for spiders. In ants, the winged reproductive castes (queens and males) were not considered in the analyses because in contrast to workers it is not clear whether they originated in the study site (123 of 16,465 individuals; 0.75%). We also excluded workers, which were too damaged to allow for species identification (0.13%). Three strictly arboreal species were likewise excluded, as they cannot be recorded in a representative way using pitfall traps. However, arboreal ants, which also have ground surface-activity, were retained [45]. Likewise, we excluded juvenile stages (1211 of 5327 individuals; 22.7%) and adult individuals of spiders, whose identification features (palpal bulbs, epigyne) were missing or destroyed (254 of 5327 individuals; 4.8%), from analyses.

### Trait data

We assigned each plant species in one of the following two groups: forest species and non-forest species according to Delarze et al. [46]. For ant species, information on habitat specificity (forest specialist, generalist and open-land species) was designated from Seifert [40] and for spider species from Hänggi et al. ([47]; S3 Table). We called spider species forest specialists when they occur in deciduous forests. Edge species were excluded from this group. For each taxonomic group, we selected a set of species traits, which we considered to influence species’ response to urbanisation-related factors (Table 1 and S3 Table).

**Table 1:** Species traits of plants, ants, and spiders.

Trait	Type	Description
**Plants**		
Life form^1^	Categorical	Macrophanerophyte; nanophanerophyte; chamaephyte; hemicryptophyte; geophyte; therophyte
Reproduction type^1^	Categorical	Sexual; mixed
Ecological strategy^1^	Categorical	Following Grime (1979): C; CR; CS; CSR; S; SR
Pollination syndrome^1^	Categorical	Insects; wind
Seed dispersal type^2^	Categorical	Zoochory; anemochory; hemerochory; autochory; hydrochory
Seed mass^1^	Continuous	Mean of seed mass (mg)
**Ants**		
Body size^3^^,^ ^4^	Continuous	Maximum of the total length of workers (mm)
Main nest stratum^4^	Categorical	Wood or litter; soil or crevices; both
Number of queens^4^	Categorical	Monogynous; oligogynous; polygynous
Main food type^4^	Categorical	Animal matter; animal matter and carbohydrates; carbohydrates; grains
**Spiders**		
Body size^5^	Continuous	Mean body size (mm) weighted by the proportion of males and females recorded in this study
Hunting mode^6^	Categorical	Web building; hunting (including active hunting and ambush)

Source

^1^ [37]

^2^ [38]

^3^ species descriptions in the taxonomic literature, sources listed under 4, and own measurements

^4^ [29] and three web-based resources (www.antwiki.org, www.ameisenwiki.de, www.antweb.org)

^5^ [33]

^6^ [39]

Data of six plant traits (life form, reproduction type, ecological strategy following Grime [48], pollination syndrome, seed dispersal type and seed mass) were obtained from the database BiolFlor [49] and Müller-Schneider [50]. We obtained trait information for ants (body size of workers, main nest stratum, queen number, main food source) from Seifert [40], three web-based resources (<www.antwiki.org>, <www.ameisenwiki.de>, <www.antweb.org>) and in a few cases from own measurements or taxonomic species descriptions (Table 1 and S3 Table). For spider species, we assembled data of body size, and hunting mode from literature [44, 51] (Table 1 and S3 Table).

### Environmental characteristics

We estimated total cover of ground vegetation in each of the plots from the vegetation survey using the Braun-Blanquet scale [39]. Canopy closure was assessed based on three photographs in each plot and determined with the pixel counting function of Adobe Photoshop (version 10.0.1).

To examine any potential influences of soil characteristics on plant diversity, three soil samples were collected in each vegetation plot using a metal cylinder (depth: 5 cm; diameter 5.05 cm; volume 100 cm^3^) in October 2014. We pooled and mixed the three soil samples of a plot and transported them to the laboratory, where they were sieved (mesh size 2 mm) and dried at 50°C for 96 h. We determined soil moisture content (%) using the fresh to dry weight ratio and assessed soil pH in distilled water (1:2.5 soil:water) [52]. We determined total soil organic matter content (SOM, %) as loss-on-ignition of oven dried soil at 750°C for 16 h [52]. We assessed total soil organic nitrogen content (orgN, %) using the standard method of Kjeldahl [53]. Finally, we determined total phosphorus content of soil (orgP, μg PO_4_^3–^ g^–1^) using the molybdenum blue method [54].

We measured biotic and abiotic environmental characteristics in the pitfall trap plots during the autumn pitfall trap survey. To assess the complexity of the vegetation structure and the amount of dead woody debris, we used a slight modification of the point intercept method [55]. In each grid of traps, we installed a transect line in the centre of the two rows. At the beginning of the transect line, we inserted a pin vertically into the ground and recorded the number of times the pin was touched by different plant specimens up to 2 m (hereafter referred to ‘vegetation structure’) and by dead woody debris on the forest floor (hereafter referred to ‘amount of dead wood’). We repeated this procedure at intervals of 1 m resulting in a total of 26 measuring points per forest site.

To assess soil and litter characteristics, we divided the trap-grid system into three sections with each including four traps. In each grid section, we collected four soil samples. We pooled and mixed them to yield a total of three soil samples per trapping grid. In the centre of each grid section, leaf litter was collected in an area measuring 20 cm × 20 cm, dried and weighed. To assess the moisture content and pH of soil and litter and soil organic matter content, we applied the same methods as described above.

Environmental factors were used to characterize the forest sites and to explain the patterns of diversity of the focal groups rather than to examine their own response to urbanisation and forest size. We assessed soil and litter variables, vegetation structure and amount of dead wood in autumn 2014. This is adequate for soil variables because soil pH, SOM, total soil organic nitrogen and total phosphorus content are relatively constant over the whole vegetation period in the forests examined [56]. For leaf litter the autumn sampling captures the year’s input. In addition to humidity also temperature can affect biodiversity or arthropod activity. We therefore measured soil temperature close to the surface (0–5 cm) hourly at the edge of the pitfall grid throughout the study period. As the study focused on the ground-surface active ants and spiders, soil surface temperature was considered to be the most appropriate measure for temperature, and air temperature higher up in the vegetation, where some species also forage can be expected to be correlated. However, due to high degrees of vandalism the temperature data were incomplete and could not be used in the models. A finer-scaled soil temperature survey conducted in nine of the forest sites, however, revealed only relatively small differences among the forests [56].

### Landscape characteristics and recreational pressure

For each forest, we derived land cover data of six landscape characteristics from satellite images (Google Earth, 2009). Around the most central sampling plot in each forest, we determined the percentage cover of built-up area and traffic infrastructure, urban green space (comprising gardens, parks and allotments), agricultural land and forest cover within radii of 200 m and 500 m using the pixel counting function of Adobe Photoshop (version 10.0.1). The percentage cover of sealed area (built-up area and traffic infrastructure) was used as a measure of the degree of urbanisation. Because the percentage cover of sealed area inter-correlated with the percentage covers of the other three landscape elements (all P < 0.008, S4 Table), we did not consider the percentage covers of these landscape elements for data analyses.

We used two different measures to estimate the impact of recreational pressure in the forest sites: (1) path density expressed as the total length of paths and forestry trails per forest site (in m/ha), and (2) the total trampled area within a forest (expressed in percentage of forest area).

### Data analyses

Statistical analyses were performed in R ver. 3.0.2 (www.r-project.org) and were carried out separately for the three taxonomic groups at the forest site level. Species richness consists of the total number of species recorded in all vegetation plots and pitfall traps, respectively, over the whole sampling period. In plants, we calculated Shannon diversity and evenness for each of the six vegetation plots separately and averaged them per forest site. In the ant and spider sampling, most of the forest sites were exposed to a variety of disturbances including vandalism, which caused the loss of several traps (72 out of 1560 traps, 4.6%; S5 Table). Therefore, we calculated sample-based rarefied species richness using the *specaccum* function in the package *vegan* in R. Due to positive correlations between observed and sample-based rarefied species richness (both, ants and spiders: r_s_ = 1.00, n = 26, P < 0.001), we only used rarefied species richness in the subsequent analyses (hereafter referred to as ‘species richness’). For ants, where numbers can be inflated when a trap is close to a nest, we used the proportion of traps in which a species was present to calculate Shannon diversity and evenness instead of abundance data. We further used number of individuals per trap (individual density) instead of abundance data to compare Shannon diversity and evenness among forest sites for spiders.

Preliminary analyses revealed correlations between the two radii of degree of urbanisation and the two measures of recreational pressure. In the vegetation plots, SOM further was positively correlated with soil orgN, while there were inter-correlations between soil and litter characteristics in the trap-grid system (soil moisture vs. litter moisture: r = 0.52, n = 26, P = 0.006; soil pH vs. litter pH: r_s_ = 0.56, n = 26, P = 0.003). Therefore, we only considered degree of urbanisation within the 500-m radius, path density and soil orgN in plants and litter moisture content and litter pH in ants and spiders in the subsequent analyses. Furthermore, the historical development of forests was confounded with forest size (see S4 Table for further details). Forest size thus could not be considered independently from the historical development of the forests.

Based on the percentage cover of sealed area in their surroundings, we classified the forests into areas with low (< 15%), medium (15–30%) or high (> 30%) degrees of urbanisation. We also divided forests into three size classes: small (< 4000 m^2^), medium-sized (4000–10,000 m^2^) or large (> 10,000 m^2^) forests (S2 Table). While these size classes also capture variations in forest history, for simplicity, we refer to these categories as forest size throughout the results section. The three size and three urbanisation classes were based on the distribution of available fragment sizes and percentages of sealed area following [56]. We considered the degree of urbanisation and forest size either as continuous variables (first approach) or as factors (second approach) in the statistical analyses to examine their potential effects on species diversity (species richness, Shannon diversity and evenness). However, because the two approaches revealed very similar results, we only present the results of the second approach.

We applied generalized linear models (GLM) with quasi-Poisson distributed errors using log-link function to examine potential effects of the degree of urbanisation, forest size and the corresponding interaction on species diversity and the percentages of forest specialists, and ANCOVA for the functional dispersion of the three taxonomic groups. We used degree of urbanisation (three classes), forest size (three classes) and shape (three classes: continuous forests (no shape index), shape index 1–1.5, shape index > 1.5) and management of forest sites (‘time since last thinning’: ≤ 3 years, 4–10 years or > 10 years ago) as factors, and path density and canopy closure as cofactors in the GLM and ANCOVA models of all three taxonomic groups. In plants, we further included soil moisture content, soil pH, soil orgN and orgP and cover of ground vegetation as cofactors in the GLM and ANCOVA models (S2 Table). For ants and spiders, we used SOM, litter moisture content and litter pH, amount of litter biomass, vegetation structure and amount of dead wood as cofactors in the GLM and ANCOVA models (S2 Table). In ants and spiders, we further tested the impact of these factors on the percentages of generalist species. All the environmental factors listed above were included into models as covariables. The models were then reduced following a stepwise procedure, which resulted in the dropping of several covariables. We performed multiple comparisons (Tukey contrasts) to compare differences among degrees of urbanisation, forest size, forest shape and time since last thinning, respectively, using the *glht* function in the *multcomp* package in R [57].

To show whether degree of urbanisation affected species composition of plants, ants and spiders, we used non-metric multidimensional scaling (NMDS) with Bray-Curtis dissimilarity measures. Data were square-root-transformed and Wisconsin double standardization was applied. This type of transformation involves standardization of species maxima, followed by relativization of sample total [58] Species, which were recorded in only one site, were excluded from the analyses.

Permutational multivariate analyses of variance (PERMANOVA) were used to test whether degree of urbanisation, forest size and local forest characteristics affected species composition of plants, ants and spiders [59]. The local forest characteristics were included as cofactors (S6 Table). For plants, soil moisture, soil pH, total soil phosphorous content, total soil nitrogen content, and ground vegetation cover were thus included in the analysis. For ants and spiders, path density, canopy closure, total soil organic matter content, litter moisture, litter pH, litter biomass, amount of dead wood, and vegetation structure were included as cofactors. For all three groups of organisms, we further included the shape index and the time since last thinning as factors. All PERMANOVA tests were based on 999 permutations of the untransformed raw data, using the *adonis* function in the vegan R-package [60].

As a measure for functional diversity we calculated functional dispersion for each taxonomic group according to Villéger et al. [61] using the *dbFD* function with Cailliez corrected distance matrices in the package *FD* in R [62]. As for the NMDS and PERMANOVA analyses, we only used those species that occurred in more than one forest site. We used ANCOVA to examine the impact of degree of urbanisation, forest size and local forest characteristics on the functional dispersion of plants, ants, and spiders.

## Results

Across the 26 forests, we recorded a total of 130 vascular plant species (30.7 species per forest, range: 17–53 species; S5 Table). Eighty-three of the 130 plant species (63.8%) were forest specialists. The most common plant species in the ground vegetation layer were *Hedera helix* and *Quercus robur*, which occurred in 26 forests and *Fraxinus excelsior* and *Geum urbanum*, which were found in 25 forests.

Overall, we collected 16,321 ants belonging to 28 species in the 26 forests examined. On average, we captured 10.0 ant species (range: 6–16 species) per forest (S5 Table). Among ant species, 10 were forest specialists or dependent on wood for their nest construction (35.7% of species found), while the reminder were habitat generalists (5 species; 17.9%) or even open-land species (13 species; 46.4%). *Myrmica rubra*, a generalist species, which is often found in urban habitats, comprised 41.7% of all ants collected. It occurred in 19 of the 26 sites, with 75.3% of individuals collected in a particular site. Six ant species were more widespread: *Myrmecina graminicola* and *Temnothorax nylanderi* (26 forests each), *Lasius niger* and *Stenamma debile* (23 each), *Lasius brunneus* (22) and *Myrmica ruginodis* (20).

We collected 5,327 spiders belonging to 109 species. On average, 18.3 spider species (range: 10–31 species) were captured per forest (S5 Table). In spiders, 30 species were forest specialists (27.5%), 57 habitat generalists (52.3%) and 21 open-land species (19.3%). The most common spider species were *Tenuiphantes flavipes* (26 forests), *Trochosa terricola* (21), *Diplostyla concolor* and *Pardosa saltans* (19 each).

### Effects of degree of urbanisation on species diversity

**Fig 2:**
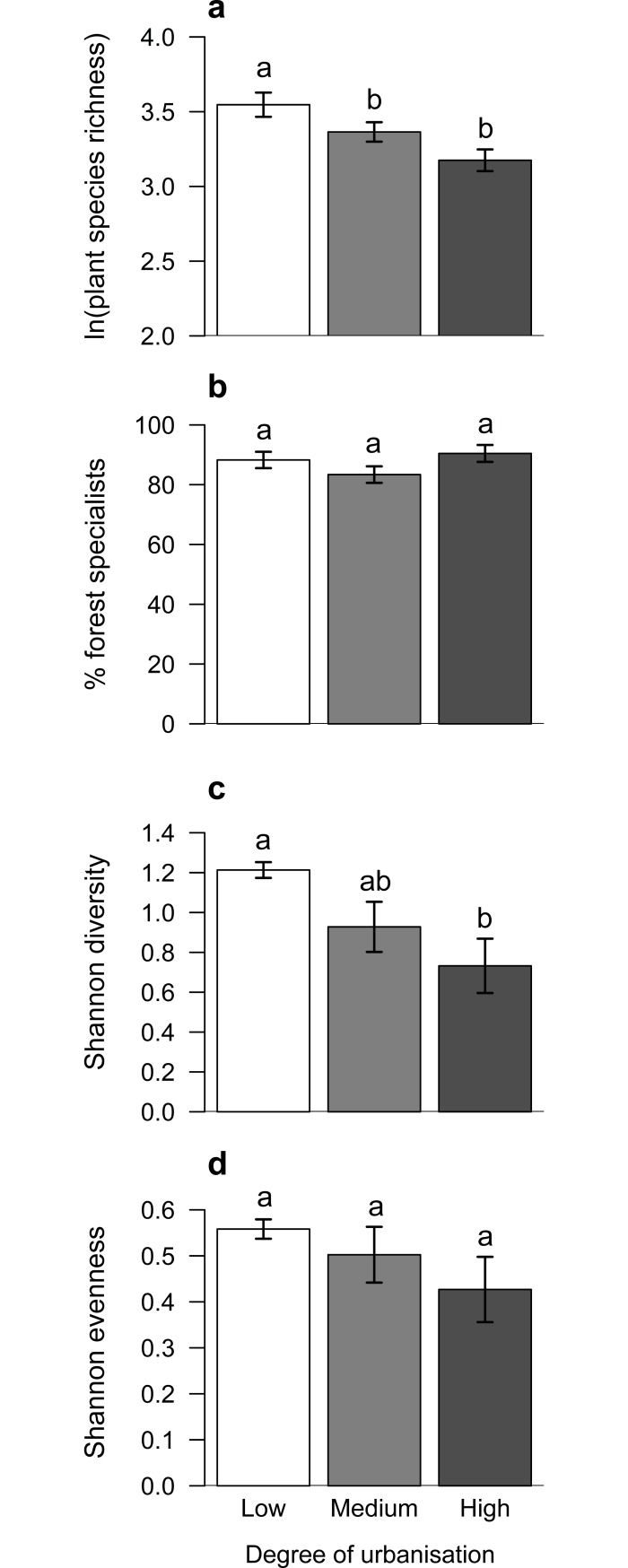
Plant species richness (a; mean ± SE), percentage of forest specialists (b), Shannon diversity (c) and evenness (d) in forests, which were located in areas with different degrees of urbanisation.

Plant species richness, the percentage of forest specialists and Shannon diversity of plants were affected by the degree of urbanisation (Table 2; Fig 2). While the species richness and Shannon diversity of plants decreased with increasing degree of urbanisation (Fig 2A and 2C), the percentage of forest specialists was slightly higher in forests located in areas with either a low or high degree of urbanisation compared to forests situated in areas with a medium degree of urbanisation (Fig 2B). Furthermore, Shannon evenness of plants tended to decrease in forests with increasing percentage cover of sealed areas in their surroundings (Fig 2D).

**Table 2:** Summary of GLM analyses examining the effects of degree of urbanisation, forest size and shape, forest management (time since last thinning), disturbance (indicated by path density), canopy closure, soil characteristics (moisture, pH, soil orgN and orgP) and cover of ground vegetation on the species richness, percentage of forest specialists, Shannon diversity and evenness of vascular plants.

	Species richness^1^			Percentage of forest specialists			Shannon diversity			Shannon evenness		
	df	F	P	df	F	P	df	F	P	df	F	P
Degree of urbanisation	2,23	8.43	**0.004**	2,23	4.59	**0.029**	2,23	7.71	**0.004**	2,23	3.44	0.056
Forest size	2,21	0.04	0.96	2,21	2.07	0.16	2,21	1.78	0.20	2,21	4.88	**0.021**
Shape index	2,19	1.43	0.27	2,19	18.59	**<0.001**	–	–	–	–	–	–
Time since last thinning	–	–	–	2,17	1.47	0.26	2,19	2.64	0.10	2,19	5.57	**0.014**
Path density	–	–	–	–	–	–	–	–	–	–	–	–
Canopy closure	1,18	7.84	**0.015**	1,16	9.53	**0.008**	–	–	–	–	–	–
Soil moisture content	–	–	–	1,15	2.91	0.11	1,18	7.83	**0.012**	1,18	9.85	**0.006**
Soil pH	–	–	–	1,14	2.63	0.13	–	–	–	–	–	–
Soil orgN^1^	–	–	–	–	–	–	1,17	1.23	0.28	1,17	1.50	0.24
Soil orgP^1^	1,17	3.47	0.085	–	–	–	–	–	–	–	–	–
Cover of ground vegetation	–	–	–	–	–	–	†	†	†	†	†	†
Degree of urbanisation*forest size	4,13	1.41	0.28	–	–	–	–	–	–	–	–	–

Significant P-values (< 0.05) are in bold

^1^ log-transformed

–Factor was excluded from the model by step-wise reduction

† Factor was not included in the model

In ants, the percentage of generalists was influenced by the degree of urbanisation, being slightly higher in forests with dense settlements in their surroundings than in forests located in areas with low or medium degrees of urbanisation (Table 3). In contrast, species richness, Shannon diversity and evenness of ants were not affected by the degree of urbanisation (Table 3).

**Table 3:** Summary of GLM analyses examining the effects of degree of urbanisation, forest size and shape, forest management (time since last thinning), disturbance (indicated by path density), canopy closure, soil organic matter content, litter characteristics (moisture, pH) and structural diversity measures (litter biomass, vegetation structure and amount of dead wood) on the species richness, percentages of forest specialists and generalists, Shannon diversity and evenness of ants.

	Sample-based rarefied species richness			Percentage of forest specialists			Percentage of habitat generalists			Shannon diversity			Shannon evenness		
	df	F	P	df	F	P	df	F	P	df	F	P	Df	F	P
Degree of urbanisation	2,23	2.94	0.083	2,23	1.84	0.21	2,23	4.31	**0.049**	2,23	3.21	0.06	2,23	0.33	0.72
Forest size	2,21	2.71	0.10	2,21	6.09	**0.018**	2,21	0.71	0.51	2,21	4.71	**0.023**	2,21	1.57	0.15
Shape index	–	–	–	2,19	1.20	0.34	2,19	2.56	0.14	–	–	–	–	–	–
Time since last thinning	–	–	–	2,17	1.83	0.21	2,17	2.59	0.13	–	–	–	–	–	–
Path density	1,20	6.68	**0.022**	–	–	–	–	–	–	1,20	7.45	**0.014**	–	–	–
Canopy closure	–	–	–	1,16	5.29	**0.044**	–	–	–	–	–	–	1,20	1.57	0.23
Soil organic matter content^1^	–	–	–	–	–	–	1,16	5.79	0**.040**	–	–	–	–	–	–
Litter moisture content	–	–	–	1,15	2.46	0.15	1,15	6.31	0**.033**	–	–	–	–	–	–
Litter pH	1,19	7.12	**0.018**	–	–	–	–	–	–	1,19	5.38	**0.032**	–	–	–
Amount of litter biomass^1^	–	–	–	–	–	–	–	–	–	–	–	–	–	–	–
Vegetation structure^1^	–	–	–	1,14	1.58	0.24	1,14	5.65	**0.041**	–	–	–	–	–	–
Amount of dead wood	1,18	1.36	0.13	–	–	–	1,13	1.61	0.24	1,18	6.47	0**.020**	–	–	–
Degree of urbanisation*forest size	4,14	1.09	0.40	4,10	2.55	0.10	4,9	1.93	0.19	–	–	–	–	–	–

Significant P-values (< 0.05) are in bold

^1^ log-transformed

–Factor/Co-factor was excluded due to by step-wise model reduction procedure

In spiders, both the percentages of forest specialists and generalists were influenced by the degree of urbanisation (Table 4). The percentage of forest specialists was lower in forests situated in areas with medium degree of urbanisation than in forests located in areas with high or low degree of urbanisation. In contrasts, the percentage of generalists was higher in forests located in areas with medium and high degrees of urbanisation than in forests surrounded by sparse settlements. However, the species richness, Shannon diversity and evenness of spiders did not differ among the urbanisation classes. We found an interaction between degree of urbanisation and forest size for the Shannon evenness of spiders: Small forests located in areas with a low degree of urbanisation had lower Shannon evenness indices than small forests located in areas with medium and high degrees of urbanisation, whereas medium-sized and large forests showed similar Shannon evenness indices in areas with different degrees of urbanisation.

**Table 4:** Summary of GLM analyses examining the effects of degree of urbanisation, forest size and shape, forest management (time since last thinning), disturbance (indicated by path density), canopy closure, soil organic matter content, litter characteristics (moisture, pH) and structural diversity measures (litter biomass, vegetation structure and amount of dead wood) on the species richness, percentages of forest specialists and generalists, Shannon diversity and evenness of spiders.

	Sample-based rarefied species richness			Percentage of forest specialists			Percentage of habitat generalists			Shannon diversity			Shannon evenness		
	df	F	P	df	F	P	df	F	P	df	F	P	df	F	P
Degree of urbanisation	2,23	0.60	0.56	2,23	9.30	**0.002**	2,23	4.48	**0.028**	2,23	1.36	0.30	2,23	1.67	0.23
Forest size	2,21	0.02	0.98	2,21	3.96	**0.039**	2,21	0.29	0.75	2,21	1.90	0.20	2,21	3.77	0.051
Shape index	–	–	–	–	–	–	–	–	–	2,19	1.33	0.31	2,19	3.38	0.066
Time since last thinning	–	–	–	–	–	–	–	–	–	2,17	1.57	0.26	–	–	–
Path density	–	–	–	1,20	2.54	0.13	1,20	4.36	0.053	–	–	–	1,18	1.94	0.19
Canopy closure	1,20	4.33	0.052	–	–	–	–	–	–	1,16	1.88	0.20	–	–	–
Soil organic matter content^1^	–	–	–	1,19	2.67	0.12	1,19	1.78	0.20	–	–	–	–	–	–
Litter moisture content	1,19	5.29	**0.034**	–	–	–	–	–	–	1,15	2.17	0.17	–	–	–
Litter pH	1,18	1.23	0.28	1,18	7.20	**0.016**	1,18	2.64	0.12	–	–	–	–	–	–
Amount of litter biomass^1^	–	–	–	1,17	4.41	0.051	1,17	1.77	0.20	–	–	–	–	–	–
Vegetation structure^1^	–	–	–	–	–	–	1,16	3.68	0.073	1,14	2.06	0.18	1,17	1.77	0.21
Amount of dead wood	–	–	–	–	–	–	–	–	–	–	–	–	–	–	–
Degree of urbanisation*forest size	–	–	–	–	–	–	–	–	–	4,10	1.51	0.27	4,13	5.20	**0.010**

Significant P-values (< 0.05) are in bold

^1^ log-transformed

–Factor was excluded from the model by step-wise reduction

### Effects of the size and shape of forests on species diversity

Shannon evenness of plants slightly increased with forest size, but was not affected by the shape of the forests (Table 2). In contrast, the percentage of forest specialists was influenced by the shape of forests (Table 2; S1 Fig), but did not differ among size classes (Table 2). A higher percentage of forest specialists was found in large continuous forests and forests with a shape index between 1.0 and 1.5 than in forests with a shape index higher than 1.5 (S1 Fig). The species richness and Shannon diversity of plants were neither influenced by the size nor shape of forests (Table 2).

In ants, the percentage of forest specialists and Shannon diversity of ants were positively related to forest size (Table 3; S1 Fig), but were not influenced by the shape of forests. The species richness of ants and percentage of generalists of ants were neither influenced by the size nor the shape of forests (Table 3).

Similar to ants, the percentage of spider forest specialists was higher in large than in medium-sized and small forests (Table 4; S1 Fig), but was not influenced by forest shape. Shannon evenness tended to be affected by forest size, being slightly higher in medium-sized than in small forests. Furthermore, Shannon evenness tended to be influenced by the shape index of forests. Large continuous forests and forests with a shape index between 1.0 and 1.5 exhibited a more even spider species distribution than forests with a shape index larger than 1.5. However, species richness, percentage of generalists and Shannon diversity of spiders were neither affected by the size nor shape of the forests (Table 4).

### Effects of forest site characteristics on species diversity measures

Plant species richness decreased with increasing canopy closure of forests (r_s_ = –0.61, n = 26, P < 0.001), while the percentage of forest specialists increased (r_s_ = 0.47, n = 26, P = 0.017; Table 2). Both Shannon diversity and evenness of plants were positively related to soil moisture content (diversity: r_s_ = 0.52, n = 26, P = 0.007; evenness: r = 0.42, n = 26, P = 0.031). Furthermore, Shannon evenness of plants was affected by the time since last thinning (Table 2). It was higher in forests, which were managed recently (≤ 3 years or 4–10 years) than in forests, which were thinned last time more than 10 years ago. However, path density, soil pH, soil orgN and orgP and the cover of ground vegetation did not influence any of the plant diversity measures examined (Table 2).

In ants, species richness was negatively affected by litter pH (r_s_ = –0.53, n = 26, P = 0.005) and tended to increase with path density (r = 0.38, n = 26, P = 0.058; Table 3). The percentage of forest specialists was influenced by canopy closure, being highest at moderate structural diversity of vegetation. The percentage of generalist ant species was positively affected by soil organic matter, litter pH, and vegetation structure (Table 3). However, the Spearman correlations for these covariables were not significant (all P > 0.2). Shannon diversity of ants tended to be positively affected by path density (r_s_ = 0.37, n = 26, P = 0.062), and negatively by litter pH (r_s_ = –0.35, n = 26, P = 0.077) and amount of dead wood (Table 3). However, the Spearman correlation for the latter was not significant. Shannon evenness of ants was not affected by any of the forest characteristics.

The species richness of spiders decreased with litter moisture content (r = –0.41, n = 26, P = 0.038) and tended to be affected by canopy closure (Table 4). However, the latter was not a linear relationship. The percentage of forest specialists was influenced by litter pH with species richness highest at intermediate values of pH (Table 4). However, none of the forest characteristics examined had a significant impact on the percentage of generalist species, and Shannon diversity and evenness of spiders (Table 4).

### Species composition

**Fig 3:**
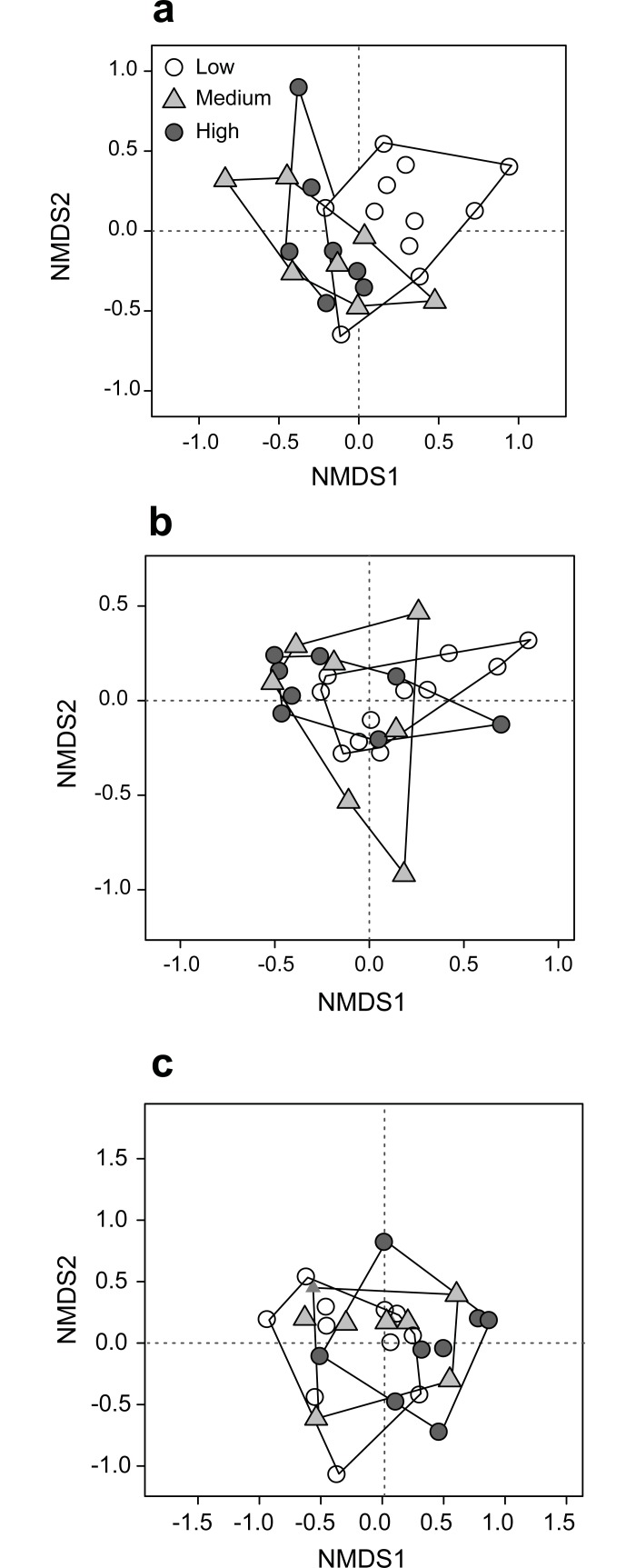
**NMDS of (a) plant, (b) ant and (c) spider species composition.** Forests sites are grouped according to their degree of urbanisation (low, medium, high).

For plants, multivariate analysis using NMDS showed that plant species composition shifted from low to high degrees of urbanisation but with some overlap (Fig 3A). PERMANOVA confirmed that plant species composition was significantly affected by forest size (F_2,19_ = 2.42, P = 0.005). However, only a marginal tendency was found for degree of urbanisation (F_2,19_ = 1.47, P = 0.099). Plant species composition was also significantly affected by soil moisture (F_1,19_ = 2.82, P = 0.014) and total soil organic nitrogen content (F_1,19_ = 3.59, P = 0.001). Some common species showed marked differences in their frequencies depending on the degree of urbanisation or forest size (S7 Table). For example, the frequency of *Arum maculatum* and *Duchesnea indica* decreased with increasing degrees of urbanisation, while *Alliaria petiolata* was most frequent at intermediate degrees of urbanisation, and *Tilia platyphyllos* was most frequent in sites with high degrees of urbanisation.

Similar to the findings for plant species composition, ant species composition showed a shift from areas with a low degree of urbanisation to those with a high degree of urbanisation, though with some overlap (Fig 3B). Moreover, PERMANOVA showed that ant species composition was significantly affected by the degree of urbanisation (F_2,15_ = 1.86, P = 0.045). Ant species composition was also affected by forest size (F_2,15_ = 2.79, P = 0.005). Furthermore, canopy cover was also significantly affecting ant species composition (F_2,15_ = 2.30, P = 0.035). While many common species were similarly often present in sites with different degrees of urbanisation or of different size, some showed marked differences (S7 Table). For example, the generalist species *Myrmica rubra* occurred in all sites with high degrees of urbanisation, but only in three quarters of sites with a medium degree of urbanisation, and in just over half of sites with a low degree of urbanisation.

Similarly, for spiders, there was a shift in species composition from highly to less urbanised areas (F_2,17_ = 2.63, P = 0.001; Fig 3C). Spider species composition was also affected by forest size (F_2,17_ = 1.62, P = 0.032). Furthermore, spider species composition was influenced by most forest characteristics examined: litter moisture content (F_1,17_ = 1.86, P = 0.028), SOM (F_1,17_ = 1.73, P = 0.049), vegetation structure (F_1,17_ = 1.72, P = 0.047), amount of dead wood (F_1,17_ = 1.98, P = 0.015). As for plants and ants, some spider species showed marked differences in their frequency of occurrence in forests with different degrees of urbanisation and of different size (S7 Table). Examples include *Histopona torpida* and *Palliduphantes pallidus*, which decreased in frequency with increasing degrees of urbanization, and *Diplostyla concolor*, which was most frequent in highly urbanized sites.

### Functional dispersion

Plant functional dispersion was affected by the degree of urbanisation (F_2,16_ = 3.92, P = 0.041) and forest size (F_2,16_ = 3.68, P = 0.049; S8 Table). Furthermore, plant functional dispersion was influenced by the time since last thinning (S8 Table).

Considering ants, functional dispersion tended to be influenced by forest size (F_2,13_ = 3.68, P = 0.054) (S9 Table). Furthermore, ant functional dispersion was significantly affected by litter moisture (F_2,13_ = 12.63, P = 0.004; S9 Table). In contrast spider functional dispersion was not significantly influenced by degree of urbanisation, forest size, or habitat characteristics (S9 Table).

## Discussion

Our results showed that the response to the degree of urbanisation and forest size considerably varied among the three taxonomic groups. However, when we grouped species according to their habitat specificity, we observed a reduction in the percentage of forest specialist species with decreasing forest size in both arthropod groups. In addition to degree of urbanisation and forest size, species diversity and species composition of plants were determined by abiotic site characteristics and those of ants and spiders by the structural diversity of both leaf litter and vegetation.

### Effect of urbanisation on species diversity

During the last decades, the worldwide urban sprawl and the subsequent destruction and isolation of green areas represent major drivers for local species extinction [3]. Hence, we expected a decrease in species diversity (species richness, Shannon diversity and evenness) with increasing degree of urbanisation. However, we only found this to be the case in plants. Cameron et al. [63] reported similar results for plant species richness, but did not find any effect on plant diversity. In contrast, McKinney [28] found the highest number of plant species in areas with medium degree of urbanisation, whereas Vallet et al. [25] did not detect any difference in total species richness of plants between urban and rural woodlands. These outcomes may be due to differences in the number of non-native plant species, the spatial dimension of the study areas and the degree of urbanisation associated with differences in habitat diversity ([28] and references therein). In our study sites we only found very few neophytes.

In ground surface-active ants and spiders, the lack of response of species diversity to urbanisation contrasted our hypothesis and the findings of other studies conducted on soil arthropods in forests, which showed either a negative (carabids: [16]), hump-shaped (spiders: [32], carabids: [64]) or positive response (spiders: [65, 66]) on species richness in relation to the degree of urbanisation. However, similar results as in our study were reported by Alaruikka et al. [64], who argued that spiders might be more affected by local site characteristics (e.g. structural diversity) than by characteristics at the landscape scale.

The higher sensitivity of plant species richness to degree of urbanisation compared to those of higher trophic rank ants and spiders did not confirm our hypothesis and contrasted findings of several multi-taxa studies (e.g. [28, 35, 67]). Comparisons with these studies, however, should be made with caution, as most of them were conducted in different habitat types and/or considered other taxonomic groups [16, 20, 35]. The taxonomic groups considered in those studies are also often closely related by showing specialised plant-herbivore interactions (e.g. [35]). Contrary to this, the majority of ant and spider species recorded in our study were food generalists and thus may better cope with the loss of some species at lower trophic rank compared to specialised herbivores or predators [23, 68], as long as primary productivity as a whole was sufficient. Another explanation for the observed pattern might be differences in mobility of the three focal groups. As plants are sessile, they are more strongly influenced by their immediate surroundings and can hardly evade unfavourable environmental conditions caused by urbanisation compared to ants and spiders. Furthermore, the seeds of most plant species recorded in the present study are dispersed by animals. Hence, negative impacts of urban sprawl on the behaviour, mobility and diversity of these seed dispersers, may have enhanced the vulnerability of plants to urbanisation.

Several urban studies reported a replacement of forest specialists by generalist species with increasing degree of urbanisation suggesting that forest specialists are more sensitive to urbanisation-related disturbances [31, 32, 65]. While this was the case in ants, plants and spiders showed the lowest percentages of habitat specialists in forests located in areas with medium degree of urbanisation (15–30% sealed area). This finding was unexpected and may be a result of combined effects of differences in habitat diversity in the surroundings, which may be highest at medium levels of urbanisation, and of refugia effects of forests in highly urbanised areas.

### Effects of forest size and shape on species diversity

As a consequence of proceeding urban development, many forest sites are characterised by intense isolation and small size. Thus, it is important to examine how habitat size affects biodiversity, and how this factor interacts with the degree of urbanisation in its surroundings. Theory of island biogeography predicts that small habitat patches contain less species than large habitat patches [69]. In this study, however, we did not find a species–area relationship for any of the three taxonomic groups examined. This result rejects our hypothesis and contrasts findings of previous studies on plants [70, 71] and web spiders [72] conducted in urban forests. Partly in line with our finding, Gibb and Hochuli [21] did not record a species–area relationship in spiders either and even reported an increase of ant species richness with decreasing forest size. Most studies, however, which failed to uncover area-related effects on species richness, were typically conducted in forests much larger (e.g. [21]: 4–80 km^2^) than those in our study.

Even though forest size did not influence total species richness, we recorded higher percentages of forest specialists of ants and spiders in large than in small and medium-sized forests. Possible explanations might be a higher proportion of edge to different habitat types in small compared to large forests and, thus, a replacement of forest specialists by generalists and open-land species [21, 73]. Indeed, we found higher percentages of open-land species in small than large forests (ants: 32.3% vs. 20.1%; spiders: 11.0% vs. 4.9%). Regarding spiders, most open-land species were hunters in this study. We suggest that they may have temporarily visited forests for foraging rather than permanently living in them. Similarly, foraging ant workers from nests outside the fragments may have visited the edge zone of small forests.

As forest size was not independent of forest history in our study, some of the observed differences in percentage forest specialists for ants and spiders may also be the result of some of the forest sites having previously been non-forested habitats. However, none of the forests were very recent in origin (all the study sites were marked as forest on old maps for at least 44–137 years), and even small fragments harboured forest specialist species. Indeed small forests were not per se less suitable habitats for forest specialists as demonstrated in plants. In our study, interestingly, the shape rather than the size/history of forests was the main predictor of the percentage of forest specialists. Forest sites, which were part of a continuous forests, and forests with a rather circular area (shape index 1–1.5) exhibited a higher percentage of forest specialists than forests with a more complex shape (shape index > 1.5). Hence, even small urban forest sites of comparably recent origin can serve as habitat for numerous forest specialists, if the proportion of edge to other habitat types and associated changes in the abiotic environment are minimized. However, most of the small forest sites in our study were dominated by a few plant species–independent of the degree of urbanisation in their surroundings.

### Effects of forest site characteristics on species diversity

Plant species richness and the percentage of forest specialists were related to canopy closure considered as a proxy for light conditions, while soil moisture content was a key predictor of Shannon diversity and evenness, highlighting the importance of abiotic site characteristics for plant diversity.

Similarly, in ants, canopy closure was important in explaining the percentage of forest specialists. Furthermore, leaf litter characteristics (litter moisture, litter pH) were important determinants for ant diversity. In urban forests, leaf litter biomass can be reduced as a result of recreational use. This would not only affect ant species with nests within this layer, but also the many species foraging there.

In our study, the majority of both spider species and individuals belonged either to the family Linyphiidae (44.0% and 57.9%), which build their webs in leaf litter and mainly low vegetation, or Lycosidae (9.2% and 22.1%), which are active hunters. Hence, we expected a strong response of spiders to changes in the structural diversity of leaf litter and vegetation. Surprisingly, these two variables had no significant role in explaining variation in overall spider diversity. This lack of response may be partly explained by the habitat specificity of spider species, since we observed a trend towards an increase in the percentage of forest specialists with the amount of leaf litter biomass. This positive relationship may be also the reason for the high percentage of forest specialists recorded in large forests, which exhibited a higher amount of leaf litter biomass (mean: 335.2 g m^-2^) than small and medium-sized forests in this study (157.6 and 138.5 g m^-2^).

### Species composition

Species composition may change even when species richness is maintained [19]. Urban communities can be a subset of the regional species pool, often biased towards generalists, which are better adjusted to a stressful environment [31, 32], or they may be novel by comprising many non-native species [3]. While we recorded few non-native species, the urban forests in this study harboured many generalist and open-land species, in line with other studies (e.g. [31, 32]). This is likely a consequence of differences in disturbance intensity and a small-scale habitat mosaic. Nevertheless, many forest specialists persisted including a few species listed as threatened for Switzerland. However, the red list for ants is out-dated and no such list exists for spiders, and we thus did not analyse threatened species separately. As our fragments were small compared to other studies on this topic (e.g. [21, 31, 71]), our findings highlight the sometimes-overlooked conservation value of even small, heavily disturbed habitats.

PERMANOVA showed that as hypothesized, groups at higher trophic rank were more strongly affected by urbanisation. While this was not the case for species richness and diversity, the shift was visible in species composition. Plant species composition did only show a weak trend towards differences among the urbanisation classes, while species composition of the predaceous spiders significantly shifted with increasing degree of urbanisation. In line with our expectation, spider species composition was more similar in highly than in less urbanised areas. Ants fell between, with highly urbanised areas having a significantly changed species composition. Most spider species are generalist predators. We expected that urbanisation might affect specialised predators or parasites in our study area even more [23]. Indeed, none of the three social parasitic ant species, which use host species to found new colonies, were present in highly urbanised forest sites, even though one was common in seven other sites. Species at high trophic rank, therefore, should receive special attention when managing urban habitats.

Interestingly, forest size was important for explaining species composition of all groups. This may have also partly reflected the effects of the history of the forest sites, as species composition may have not reached equilibrium yet in sites that had been previously non-forested habitats, or in forest fragments whose area has been reduced. This may also have affected some local environmental conditions such as soil-related factors, alongside current effects such as disturbance and forest management. However, none of the forests were very recent in origin. Local abiotic factors (soil moisture and soil orgN) were important drivers for plants species composition. In contrast, only canopy closure helped to explain ant species composition, while spider species composition was affected by both abiotic and structural forest characteristics. These results mirror the importance of local abiotic habitat characteristics as key drivers for plant species diversity measures. Combining results for species composition and diversity we find that both abiotic and structural forest characteristics are important in explaining arthropod diversity and species composition. Structural forest characteristics may be a surrogate for food availability. However, we did not directly measure food availability for arthropods, though e.g. SOM may be related to it, as it supports detrivores and thus potential prey [74, 75]. This finding indicates opportunities to increase the conservation values of urban forests, because local site characteristics are more amenable to management efforts than landscape factors.

### Functional dispersion

It is expected that functional dispersion should decrease with increasing urbanisation because of an enhanced influence of environmental filtering in stressful urban environments. Some species fulfil unique roles, while others have similar functions within an ecosystem. Thus, local species loss or shifts in relative abundance can reduce the abundance and efficiency of functional traits in niche space and subsequently ecosystem functioning [76]. The observed changes in species composition in our study should thus translate to changes in functional diversity [61]. Indeed we observed that functional dispersion of plants decreased with increasing degrees of urbanisation. That this decrease in functional dispersion was a result of an increasingly stressful environment, was also supported by the finding that functional dispersion decreased with forest size. Small fragments with a high proportion of edge habitat were assumed to be exposed to most stress.

In contrast to the situation found for plants, functional dispersion in the two arthropod groups was not influenced by these two main factors, with only that of ants showing a non-significant trend to be affected by forest size. Neither did functional dispersion change depending on most of the local environmental factors examined. Given the results from the PERMANOVAs, we would have expected the observed shifts in species composition to result in larger effects on functional dispersion also for the ground surface-active arthropod community. For example, the litter layers in some urban forest fragments were reduced as a consequence of the high levels of disturbance, which could have been expected to reduce habitat quality and thus the presence of functional groups associated with leaf litter.

## Conclusions

Our results showed that species richness of the taxonomic groups was not an ideal indicator of biodiversity change in urban landscapes, as it masked shifts in species composition and relative abundance of species with different habitat specificity. Using a multi-taxa approach, we further found that the effect of urbanisation on species composition increased with trophic rank. This highlights the necessity to consider different taxonomic and functional groups in urban planning to maximize conservation value of urban green areas. In the short term, urban planners could focus on small-scale environmental factors, which proved to be important determinants of species diversity and species composition. For example, protection of litter layers and ground vegetation could be enhanced using simple management practices. However, the influence of large-scale factors like the proportion of sealed area in the surroundings and forest size on forest specialists indicates that also more complex changes at the landscape level are essential to maintain vulnerable elements of forest communities.

## Supporting information

S1 Fig Forest specialists in relation to size and shape of urban forests. Percentage forest specialist species of a) ants and b) spiders in fragments of different size; size classes are small (< 4000 m^2^), medium-sized (4000–10,000 m^2^) and large (> 10,000 m^2^); and c) percentage of forest specialist species of plants depending on the shape of the fragment. The shape index was calculated following Gyenizse et al. [28]. A shape index of 1 corresponds to a circular area, which is considered as most stable and resistant against biotic and abiotic effects from the surrounding landscape. Classes are A: continuous forest, B: shape index between 1 and 1.5, C: shape index > 1.5.
(DOCX)

S1 Table Description of forest sites. Characteristics of the 26 forests examined in Basel (Switzerland) and its surroundings.
(DOCX)

S2 Table Landscape, forest and plot characteristics recorded during field surveys. (XLS)

S3 Table Species and trait lists. Species list of (a) vascular plants, (b) ants and (c) spiders. Habitat specificity, conservation status (Red List) and a set of traits, which we considered to influence species’ response to urbanisation-related factors are shown. Traits not used for analyses are in parentheses.
(DOCX)

S4 Table Correlations within and among landscape and site characteristics. Results of Pearson’s (r) and Spearman’s rank (r_s_) correlation, Contingency table (χ^2^-test) and Kruskal-Wallis test examining the relationship between observed species richness and rarefied species richness (a) and among landscape and forest characteristics for all three taxonomic groups (b), in the vegetation plots (c) and in the trap-grid system (d)
(DOCX)

S5 Table Species–site matrices for plants, ants, and spiders. For the arthropod survey, the number of recollected traps per forest site is presented.
(XLSX)

S6 Table Data used to perform PERMANOVA. (XLSX)

S7 Table Percentage of sites in which common species occur for different degrees of urbanisation and forest size classes. Common species are defined as occurring in at least 10 of the sites. Means are given for less common species.
(XLS)

S8 Table Functional dispersion: Summary of ANCOVAs of plants. Summary of ANCOVAs examining the effects of degree of urbanisation, forest size and shape, forest management (time since last thinning), disturbance (indicated by path density), canopy closure, soil characteristics (moisture, pH, soil organic nitrogen (orgN) and phosphorus (orgP) content) and cover of ground vegetation on functional dispersion of vascular plants.
(DOCX)

S9 Table Functional dispersion: Summary of ANCOVAs of ants and spiders. Summary of ANCOVAs examining the effects of degree of urbanisation, forest size and shape, forest management (time since last thinning), disturbance (indicated by path density), canopy closure, soil organic matter content, litter characteristics (moisture, pH) and structural diversity measures (litter biomass, vegetation structure and amount of dead wood) on functional dispersion of ants and spiders.
(DOCX)
