# Supplementary material for: Diverse effects of degree of urbanisation and forest size on species richness and functional diversity of plants, and ground surface-active ants and spiders
Source: PLoS One. 2018 Jun 19;13(6):e0199245. doi: 10.1371/journal.pone.0199245 (PMC6007905; doi:10.1371/journal.pone.0199245)
Supplement: S1 Fig — Percentage forest specialist species of a) ants and b) spiders in fragments of different size; size classes are small (< 4000 m2), medium-sized (4000–10,000 m2) and large (> 10,000 m2); and c) percentage of forest specialist species of plants depending on the shape of the fragment. The shape index was calculated following Gyenizse et al. [28]. A shape index of 1 corresponds to a circular area, which is considered as most stable and resistant against biotic and abiotic effects from the surrounding landscape. Classes are A: continuous forest, B: shape index between 1 and 1.5, C: shape index > 1.5. (DOCX) [file pone.0199245.s001.docx]

**S1 Fig. Forest specialists in relation to size and shape of urban forests.** Percentage forest specialist species of a) ants and b) spiders in fragments of different size; size classes are small (< 4000 m^2^), medium-sized (4000–10,000 m^2^) and large (> 10,000 m^2^); and c) percentage of forest specialist species of plants depending on the shape of the fragment. The shape index was calculated following Gyenizse et al. [28]. A shape index of 1 corresponds to a circular area, which is considered as most stable and resistant against biotic and abiotic effects from the surrounding landscape. Classes are A: continuous forest, B: shape index between 1 and 1.5, C: shape index > 1.5.
