# Supplementary material for: Diverse effects of degree of urbanisation and forest size on species richness and functional diversity of plants, and ground surface-active ants and spiders
Source: PLoS One. 2018 Jun 19;13(6):e0199245. doi: 10.1371/journal.pone.0199245 (PMC6007905; doi:10.1371/journal.pone.0199245)
Supplement: S1 Table — Characteristics of the 26 forests examined in Basel (Switzerland) and its surroundings. (DOCX) [file pone.0199245.s002.docx]

**S1 Table. Description of forest sites.** Characteristics of the 26 forests examined in Basel (Switzerland) and its surroundings.

|  |  |  |  |  |  |  | |  | |  | |  |  | |  |  | | |  | |  | |
| --- | --- | --- | --- | --- | --- | --- | --- | --- | --- | --- | --- | --- | --- | --- | --- | --- | --- | --- | --- | --- | --- | --- |
| Forest | Coordinates |  | Historical development | Elevation (m a.s.l.) | Exposure | Area (m^2^) | |  | Shape index | % cover of sealed area  (r = 200 m) | |  | % cover of sealed area  (r = 500 m) | |  | Time since last thinning (in y) | |  | Path density (m/ha) | | Mean canopy closure (in %) | |
|  |  |  |  |  |  |  | |  |  |  | |  |  | |  |  | |  |  |  |  |  |
| BS1 | 47° 34' 34.58" N  7° 36' 19.23" E |  | Forest | 269 | – | 21000 |  |  | Continuous | 4 |  |  | 20 |  |  | 2 |  |  | 360.6 |  | 97.81 |  |
| BS2 | 47° 34' 20.76" N  7° 36' 52.84" E |  | Planted | 263 | – | 620 |  |  | 1.25 | 22 |  |  | 22 |  |  | 7 |  |  | 580.6 |  | 98.18 |  |
| BS3 | 47° 34' 20.65" N  7° 37' 11.82" E |  | Planted | 265 | – | 4576 |  |  | 1.64 | 10 |  |  | 12 |  |  | 9 |  |  | 243.2 |  | 97.39 |  |
| BS4 | 47° 33' 45.65" N  7° 37' 44.02" E |  | Planted | 262 | S | 7458 |  |  | 3.39 | 12 |  |  | 19 |  |  | > 20 |  |  | 311.6 |  | 94.67 |  |
| BS5 | 47° 33' 12.30" N  7° 36' 51.09" E |  | Planted | 262 | NE | 7049 |  |  | 2.26 | 39 |  |  | 39 |  |  | 3 |  |  | 283.7 |  | 92.47 |  |
| BS6 | 47° 32' 43.72" N  7° 36' 26.85" E |  | Planted | 276 | – | 1084 |  |  | 1.20 | 69 |  |  | 70 |  |  | 14 |  |  | 48.0 |  | 97.80 |  |
| BS7 | 47° 32' 8.70" N  7° 35' 23.09" E |  | Fragment | 326 | E | 3009 |  |  | 2.91 | 32 |  |  | 30 |  |  | 1 |  |  | 254.6 |  | 94.63 |  |
| BS8 | 47° 32' 17.69" N  7° 35' 38.71" E |  | Planted | 303 | N | 2285 |  |  | 1.42 | 34 |  |  | 43 |  |  | 3 |  |  | 337.0 |  | 96.02 |  |
| BS9 | 47° 32' 12.27" N  7° 36' 5.97" E |  | Fragment | 321 | NE | 3244 |  |  | 1.22 | 39 |  |  | 54 |  |  | 8 |  |  | 334.8 |  | 96.77 |  |
| BS10 | 47° 31' 57.55" N  7° 36' 11.58" E |  | Fragment | 338 | NW | 14000 |  |  | 1.29 | 29 |  |  | 44 |  |  | 6 |  |  | 445.9 |  | 98.26 |  |
| BS11 | 47° 31' 47.76" N  7° 35' 49.19" E |  | Fragment | 370 | SE | 21000 |  |  | 1.32 | 19 |  |  | 23 |  |  | 9 |  |  | 410.3 |  | 95.44 |  |
| BS12 | 47° 31' 26.26" N  7° 35' 33.76" E |  | Fragment | 314 | – | 19400 |  |  | 2.12 | 9 |  |  | 14 |  |  | 4 |  |  | 319.1 |  | 94.99 |  |
| BS13 | 47° 31' 46.44" N  7° 36' 12.24" E |  | Planted | 376 | NW | 3633 |  |  | 2.35 | 28 |  |  | 46 |  |  | 16 |  |  | 715.7 |  | 98.28 |  |

**S1 Table** *continued*

|  |  |  |  |  |  |  | |  |  |  | | |  | |  |  | |  |  |  |  |
| --- | --- | --- | --- | --- | --- | --- | --- | --- | --- | --- | --- | --- | --- | --- | --- | --- | --- | --- | --- | --- | --- |
| Forest | Coordinates |  | Historical development | Elevation (m a.s.l.) | Exposure | Area (m^2^) | |  | Shape index | % cover of sealed area  (r = 200 m) | |  | % cover of sealed area  (r = 500 m) | |  | Time since last thinning (in y) | | Path density (m/ha) | | Mean canopy closure (in %) | |
|  |  |  |  |  |  |  | |  |  |  | |  |  | |  |  | |  |  |  |  |
| BS14 | 47° 35' 33.14” N  7° 40' 41.10” E |  | Forest | 473 | SW | 34000 |  |  | Continuous | 1 |  |  | 3 |  |  | > 20 |  | 23 |  | 96.19 |  |
| BS15 | 47° 35' 15.09” N  7° 40' 2.90” E |  | Planted | 346 | S | 2800 |  |  | 2.81 | 6 |  |  | 10 |  |  | 7 |  | 114.3 |  | 97.38 |  |
| BS16 | 47° 35' 5.71” N  7° 40' 9.23” E |  | Planted | 330 | S | 4061 |  |  | 1.88 | 9 |  |  | 8 |  |  | 3 |  | 448.2 |  | 97.28 |  |
| BS17 | 47° 35' 18.09” N  7° 38' 52.08” E |  | Planted | 273 | – | 258 |  |  | 1.12 | 3 |  |  | 17 |  |  | > 20 |  | 658.9 |  | 92.54 |  |
| BS18 | 47° 34' 30.78” N  7° 40' 35.28” E |  | Forest | 487 | – | 14000 |  |  | Continuous | 1 |  |  | 4 |  |  | 1 |  | 80 |  | 92.11 |  |
| BS19 | 47° 34' 25.90" N  7° 39' 53.22" E |  | Forest | 450 | NW | 50000 |  |  | 1.10 | 7 |  |  | 10 |  |  | > 20 |  | 151.4 |  | 95.58 |  |
| BS20 | 47° 34' 29.73" N  7° 39' 29.56" E |  | Fragment | 384 | NW | 36000 |  |  | 1.24 | 13 |  |  | 12 |  |  | 11 |  | 557.6 |  | 97.86 |  |
| BS21 | 47° 34' 51.72" N  7° 39' 37.41" E |  | Fragment | 302 | – | 4686 |  |  | 1.19 | 8 |  |  | 16 |  |  | 15 |  | 200.6 |  | 94.50 |  |
| BS22 | 47° 34' 53.45" N  7° 38' 51.87" E |  | Planted | 283 | – | 5765 |  |  | 1.14 | 43 |  |  | 33 |  |  | 16 |  | 582.2 |  | 97.99 |  |
| BS23 | 47° 34' 0.42" N  7° 38' 22.88" E |  | Planted | 277 | – | 4234 |  |  | 1.79 | 24 |  |  | 24 |  |  | 1 |  | 571.6 |  | 92.03 |  |
| BS24 | 47° 33' 58.73" N  7° 38' 46.35" E |  | Planted | 309 | S | 1500 |  |  | 2.64 | 24 |  |  | 14 |  |  | 1 |  | 0 |  | 94.23 |  |
| BS25 | 47° 33' 50.63" N  7° 38' 42.82" E |  | Forest | 319 | NW | 4034 |  |  | Continuous | 8 |  |  | 12 |  |  | 1 |  | 210.7 |  | 95.22 |  |
| BS26 | 47° 34' 7.38" N  7° 39' 4.87" E |  | Forest | 363 | NW | 8908 |  |  | Continuous | 10 |  |  | 12 |  |  | 2 |  | 264.6 |  | 92.32 |  |
