# Supplementary material for: Diverse effects of degree of urbanisation and forest size on species richness and functional diversity of plants, and ground surface-active ants and spiders
Source: PLoS One. 2018 Jun 19;13(6):e0199245. doi: 10.1371/journal.pone.0199245 (PMC6007905; doi:10.1371/journal.pone.0199245)
Supplement: S3 Table — Species list of (a) vascular plants, (b) ants and (c) spiders. Habitat specificity, conservation status (Red List) and a set of traits, which we considered to influence species’ response to urbanisation-related factors are shown. Traits not used for analyses are in parentheses. (DOCX) [file pone.0199245.s004.docx]

**S3 Table. Species and trait lists.** Species list of (a) vascular plants, (b) ants and (c) spiders. Habitat specificity, conservation status (Red List) and a set of traits, which we considered to influence species’ response to urbanisation-related factors are shown. Traits not used for analyses are in parentheses.

a) Vascular plants

|  |  |  |  |  |  |  |  |  | |
| --- | --- | --- | --- | --- | --- | --- | --- | --- | --- |
| **Species** | **Habitat specificity^1^** | **Red List^2^** | **Plant life form^3^** | **Reproduction type^3^** | **Ecological strategy^3, 4^** | **Pollination syndrome^3^** | **Seed dispersal type^5^** | **Mean seed mass (mg)^3^** | |
|  |  |  |  |  |  |  |  |  |  |
| *Acer campestre* L. | Forest species | LC | Macrophanerophyte | Mixed | C | Insects | Anemochory | 96.8 |  |
| *Acer platanoides* L. | Forest species | LC | Macrophanerophyte | Sexual | C | Insects | Anemochory | 137.2 |  |
| *Acer pseudoplatanus* L. | Forest species | LC | Macrophanerophyte | Sexual | C | Insects | Anemochory | 110.7 |  |
| *Aegopodium podagraria* L. | Forest species | LC | Hemicryptophyte | Mixed | C | Insects | Hemerochory | 2.2 |  |
| *Aesculus hippocastanum* L. | Forest species | LC | Macrophanerophyte | Sexual | C | Insects | Zoochory | 10612 |  |
| *Agrostis capillaris* L. | Non-forest species | LC | Hemicryptophyte | Mixed | CSR | Wind | Anemochory | 0.1 |  |
| *Agrostis gigantea* Roth | Forest species | LC | Hemicryptophyte | Mixed | C | Wind | Anemochory | 0.1 |  |
| *Ailanthus altissima* (Mill.) Swingle | Forest species | LC | Macrophanerophyte | Mixed | C | Insects | Anemochory | 33.6 |  |
| *Alliaria petiolata* (M. Bieb.) Cavara & Grande | Non-forest species | LC | Hemicryptophyte | Sexual | CR | Insects | Anemochory | 2.3 |  |
| *Allium* sp. | – | LC | – | – | – | Insects | – | – |  |
| *Allium ursinum* L. | Forest species | LC | Geophyte | Mixed | CSR | Insects | Zoochory | 7.3 |  |
| *Anemone nemorosa* L. | Forest species | LC | Geophyte | Mixed | CSR | Insects | Zoochory | 2.6 |  |
| *Arum maculatum* L. | Forest species | LC | Geophyte | Mixed | CSR | Insects | Zoochory | 41.0 |  |
| *Berberis julianae* C.K. Schneid. | Non-forest species | LC | Nanophanerophyte | – | – | Insects | Zoochory | – |  |
| *Berberis vulgaris* L. | Non-forest species | LC | Nanophanerophyte | Sexual | C | Insects | Zoochory | 11.9 |  |
| *Borago officinalis* L. | Non-forest species | LC | Hemicryptophyte | Sexual | CR | Insects | Zoochory | 15.5 |  |
| *Brachypodium pinnatum* aggr. | Forest species | LC | Hemicryptophyte | Mixed | CS | Wind | Anemochory | 2.8 |  |
| *Brachypodium sylvaticum* (Huds.) P. Beauv. | Forest species | LC | Hemicryptophyte | Mixed | CS | Wind | Anemochory | 0.6 |  |
| *Buxus sempervirens* L. | Forest species | NT | Nanophanerophyte | Sexual | CS | Insects | Anemochory | – |  |
| *Calystegia sepium* (L.) R. Br. | Non-forest species | LC | Geophyte | Mixed | C | Insects | Autochory | 31.3 |  |
| *Carex muricata* aggr. | – | LC | Hemicryptophyte | Mixed | C | Wind | Zoochory | 2.1 |  |

|  |  |  |  |  |  |  |  |  |  |
| --- | --- | --- | --- | --- | --- | --- | --- | --- | --- |
| **Species** | **Habitat specificity^1^** | **Red List^2^** | **Plant life form^3^** | **Reproduction type^3^** | **Ecological strategy^3, 4^** | **Pollination syndrome^3^** | **Seed dispersal type^5^** | **Mean seed mass (mg)^3^** | |
|  |  |  |  |  |  |  |  |  |  |
| *Carex pendula* Huds. | Forest species | LC | Hemicryptophyte | Mixed | CS | Wind | Anemochory | 0.8 |  |
| *Carex remota* L. | Forest species | LC | Hemicryptophyte | Mixed | CS | Wind | Anemochory | 0.3 |  |
| *Carex sylvatica* Huds. | Forest species | LC | Hemicryptophyte | Mixed | CSR | Wind | Zoochory | 1.4 |  |
| *Carpinus betulus* L. | Forest species | LC | Macrophanerophyte | Mixed | C | Wind | Anemochory | 63.8 |  |
| *Castanea sativa* Mill. | Forest species | LC | Macrophanerophyte | Sexual | C | Wind | Zoochory | – |  |
| *Circaea lutetiana* L. | Forest species | LC | Geophyte | Mixed | CS | Insects | Zoochory | 2.0 |  |
| *Clematis vitalba* L. | Forest species | LC | Nanophanerophyte | Sexual | C | Insects | Anemochory | 2.8 |  |
| *Cornus sanguinea* L. | Forest species | LC | Nanophanerophyte | Mixed | C | Insects | Zoochory | 35.2 |  |
| *Corylus avellana* L. | Forest species | LC | Nanophanerophyte | Mixed | C | Wind | Zoochory | 1042.0 |  |
| *Cotoneaster integerrimus* Medik. | Forest species | LC | Nanophanerophyte | Mixed | C | Insects | Zoochory | 9.7 |  |
| *Cotoneaster tomentosus* Lindl. | Non-forest species | LC | Nanophanerophyte | Mixed | C | Insects | Zoochory | 13.3 |  |
| *Crataegus laevigata* (Poir.) DC. | Forest species | LC | Nanophanerophyte | Sexual | C | Insects | Zoochory | 40.8 |  |
| *Crataegus monogyna* Jacq. | Non-forest species | LC | Nanophanerophyte | Sexual | C | Insects | Zoochory | 64.9 |  |
| *Dactylis glomerata* L. | Non-forest species | LC | Hemicryptophyte | Mixed | C | Wind | Anemochory | 0.6 |  |
| *Dactylis polygama* Horv. | Forest species | NT | Hemicryptophyte | Sexual | CS | Wind | Anemochory | – |  |
| *Daphne laureola* L. | Forest species | LC | Nanophanerophyte | Sexual | C | Insects | Zoochory | 103.4 |  |
| *Dryopteris filix-mas* (L.) Schott | Forest species | LC | Hemicryptophyte | Mixed | CS | Wind | Anemochory | – |  |
| *Duchesnea indica* (Andrews) Focke | Non-forest species | LC | Hemicryptophyte | Mixed | CSR | Insects | Zoochory | 0.4 |  |
| *Epilobium montanum* L. | Forest species | LC | Hemicryptophyte | Mixed | CS | Insects | Anemochory | 0.1 |  |
| *Epilobium parviflorum* Schreb. | Non-forest species | LC | Hemicryptophyte | Mixed | CS | Insects | Anemochory | 0.1 |  |
| *Euonymus europaeus* L. | Forest species | LC | Nanophanerophyte | Sexual | C | Insects | Zoochory | 33.0 |  |
| *Euphorbia amygdaloides* L. | Forest species | LC | Chamaephyte | Mixed | CS | Insects | Zoochory | 4.6 |  |
| *Fagus sylvatica* L. | Forest species | LC | Macrophanerophyte | Sexual | C | Wind | Zoochory | 254.0 |  |
| *Festuca ovina* aggr. | Non-forest species | LC | Hemicryptophyte | Sexual | CSR | Wind | Anemochory | 0.3 |  |
| *Festuca rubra* aggr. | Non-forest species | LC | Hemicryptophyte | Mixed | C | Wind | Anemochory | 0.8 |  |
| *Filipendula ulmaria* (L.) Maxim. | Forest species | LC | Hemicryptophyte | Mixed | C | Insects | Anemochory | 0.7 |  |

|  |  |  |  |  |  |  |  |  |  |
| --- | --- | --- | --- | --- | --- | --- | --- | --- | --- |
| **Species** | **Habitat specificity^1^** | **Red List^2^** | **Plant life form^3^** | **Reproduction type^3^** | **Ecological strategy^3, 4^** | **Pollination syndrome^3^** | **Seed dispersal type^5^** | **Mean seed mass (mg)^3^** | |
|  |  |  |  |  |  |  |  |  |  |
| *Fragaria vesca* L. | Forest species | LC | Hemicryptophyte | Mixed | CSR | Insects | Zoochory | 0.3 |  |
| *Fraxinus excelsior* L. | Forest species | LC | Macrophanerophyte | Sexual | C | Wind | Anemochory | 77.4 |  |
| *Galeopsis tetrahit* L. | Forest species | LC | Therophyte | Sexual | CR | Insects | Zoochory | 4.6 |  |
| *Galium mollugo* aggr. | Non-forest species | LC | Hemicryptophyte | Sexual | C | Insects | Zoochory | 0.5 |  |
| *Galium odoratum* (L.) Scop. | Forest species | LC | Geophyte | Mixed | S | Insects | Zoochory | 8.2 |  |
| *Galium spurium* L. | Non-forest species | VU | Therophyte | Sexual | CR | Insects | Zoochory | 2.7 |  |
| *Geranium robertianum* L. s.l. | Forest species | LC | Therophyte | Sexual | CSR | Insects | Autochory | 1.1 |  |
| *Geum urbanum* L. | Forest species | LC | Hemicryptophyte | Mixed | CSR | Insects | Zoochory | 2.4 |  |
| *Glechoma hederacea* L. s.l. | Non-forest species | LC | Hemicryptophyte | Mixed | CSR | Insects | Zoochory | 0.7 |  |
| *Hedera helix* L. | Forest species | LC | Nanophanerophyte | Mixed | CS | Insects | Zoochory | 20.4 |  |
| *Helictotrichon pubescens* (Huds.) Pilg. | Non-forest species | LC | Hemicryptophyte | Mixed | C | Wind | Anemochory | 1.9 |  |
| *Helleborus foetidus* L. | Forest species | LC | Chamaephyte | Sexual | CS | Insects | Zoochory | 11.4 |  |
| *Heracleum sphondylium* L. s.l. | Non-forest species | LC | Hemicryptophyte | Mixed | C | Insects | Anemochory | 5.9 |  |
| *Hypericum hirsutum* L. | Forest species | LC | Hemicryptophyte | Sexual | C | Insects | Anemochory | 0.1 |  |
| *Hypericum perforatum* L. s.l. | Non-forest species | LC | Hemicryptophyte | Mixed | C | Insects | Anemochory | 0.1 |  |
| *Ilex aquifolium* L. | Forest species | LC | Macrophanerophyte | Mixed | C | Insects | Zoochory | 140.0 |  |
| *Impatiens parviflora* DC. | Forest species | LC | Therophyte | Sexual | SR | Insects | Autochory | 7.4 |  |
| *Juglans regia* L. | Forest species | LC | Macrophanerophyte | Sexual | C | Wind | Zoochory | 6500.0 |  |
| *Lamium galeobdolon* subsp*. argentatum* (Smejkal) J. Duvign. | – | LC | Chamaephyte | Mixed | CS | Insects | Zoochory | – |  |
| *Lamium galeobdolon* subsp. *montanum* (Pers.) Hayek | Forest species | LC | Chamaephyte | Mixed | CS | Insects | Zoochory | 1.8 |  |
| *Lathyrus vernus* (L.) Bernh. s.l. | Forest species | LC | Geophyte | Mixed | CSR | Insects | Autochory | 15.2 |  |
| *Leontodon hispidus* L. s.l. | Non-forest species | LC | Hemicryptophyte | Mixed | CSR | Insects | Anemochory | 0.8 |  |
| *Ligustrum vulgare* L. | Forest species | LC | Nanophanerophyte | Sexual | C | Insects | Zoochory | 20.4 |  |
| *Lolium multiflorum* Lam. | Non-forest species | LC | Hemicryptophyte | Sexual | C | Wind | Anemochory | 2.4 |  |
| *Lonicera henryi* Hemsl. | Forest species | LC | Nanophanerophyte | – | – | Insects | Zoochory | – |  |

|  |  |  |  |  |  |  |  |  |  |
| --- | --- | --- | --- | --- | --- | --- | --- | --- | --- |
| **Species** | **Habitat specificity^1^** | **Red List^2^** | **Plant life form^3^** | **Reproduction type^3^** | **Ecological strategy^3, 4^** | **Pollination syndrome^3^** | **Seed dispersal type^5^** | **Mean seed mass (mg)^3^** | |
|  |  |  |  |  |  |  |  |  |  |
| *Lonicera pileata* Oliv. | – | LC | Nanophanerophyte | – | – | Insects | Zoochory | – |  |
| *Lonicera xylosteum* L. | Forest species | LC | Nanophanerophyte | Sexual | C | Insects | Zoochory | – |  |
| *Luzula sylvatica* (Huds.) Gaudin | Forest species | LC | Hemicryptophyte | Mixed | C | Wind | Zoochory | 0.7 |  |
| *Mahonia aquifolium* (Pursh) Nutt. | Forest species | LC | Nanophanerophyte | Mixed | C | Insects | Zoochory | 9.3 |  |
| *Maianthemum bifolium* (L.) F.W. Schmidt | Forest species | LC | Geophyte | Mixed | S | Insects | Zoochory | 11.7 |  |
| *Medicago lupulina* L. | Non-forest species | LC | Therophyte | Mixed | CSR | Insects | Zoochory | 1.6 |  |
| *Melica nutans* L. | Forest species | LC | Hemicryptophyte | Mixed | CS | Wind | Anemochory | 2.0 |  |
| *Melittis melissophyllum* L. | Forest species | LC | Hemicryptophyte | Mixed | C | Insects | Zoochory | – |  |
| *Origanum vulgare* L. | Non-forest species | LC | Hemicryptophyte | Mixed | CSR | Insects | Anemochory | 0.1 |  |
| *Paris quadrifolia* L. | Forest species | LC | Geophyte | Mixed | CSR | Insects | Zoochory | 4.0 |  |
| *Phyllitis scolopendrium* (L.) Newman | Forest species | LC | Hemicryptophyte | Sexual | CS | Wind | Anemochory | – |  |
| *Phyteuma spicatum* L. | Forest species | LC | Hemicryptophyte | Sexual | CSR | Insects | Anemochory | 0.2 |  |
| *Picea abies* (L.) H. Karst. | Forest species | LC | Macrophanerophyte | Sexual | C | Wind | Zoochory | 7.2 |  |
| *Plantago lanceolata* L. | Non-forest species | LC | Hemicryptophyte | Mixed | CSR | Wind | Zoochory | 1.8 |  |
| *Poa pratensis* L. | Non-forest species | LC | Hemicryptophyte | Mixed | C | Wind | Zoochory | 0.3 |  |
| *Poa trivialis* L. | Non-forest species | LC | Hemicryptophyte | Mixed | CSR | Wind | Zoochory | 0.1 |  |
| *Polygonatum multiflorum* (L.) All. | Forest species | LC | Geophyte | Mixed | CSR | Insects | Zoochory | 22.1 |  |
| *Potentilla reptans* L. | Non-forest species | LC | Hemicryptophyte | Mixed | CSR | Insects | Zoochory | 0.3 |  |
| *Potentilla* sp. | – | LC | – | – | – | Insects | – | – |  |
| *Primula elatior* (L.) L. | Forest species | LC | Hemicryptophyte | Mixed | CSR | Insects | Anemochory | 0.9 |  |
| *Prunus avium* L. | Forest species | LC | Macrophanerophyte | Sexual | C | Insects | Zoochory | 170.0 |  |
| *Prunus domestica* L. | – | LC | Macrophanerophyte | Mixed | C | Insects | Zoochory | – |  |
| *Prunus laurocerasus* L. | Forest species | LC | – | – | – | Insects | Zoochory | – |  |
| *Prunus padus* L. s.l. | Forest species | LC | Macrophanerophyte | Mixed | C | Insects | Zoochory | 50.0 |  |
| *Prunus serotina* Ehrh. | Forest species | LC | Macrophanerophyte | Mixed | C | Insects | Zoochory | – |  |
| *Prunus spinosa* L. | Non-forest species | LC | Nanophanerophyte | Mixed | C | Insects | Zoochory | 145.2 |  |
|  |  |  |  |  |  |  |  |  |  |
|  |  |  |  |  |  |  |  |  |  |
| **Species** | **Habitat specificity^1^** | **Red List^2^** | **Plant life form^3^** | **Reproduction type^3^** | **Ecological strategy^3, 4^** | **Pollination syndrome^3^** | **Seed dispersal type^5^** | **Mean seed mass (mg)^3^** | |
|  |  |  |  |  |  |  |  |  |  |
| *Pulmonaria officinalis* aggr. | Forest species | LC | Hemicryptophyte | Mixed | CSR | Insects | Zoochory | – |  |
| *Quercus petraea* Liebl. | Forest species | LC | Macrophanerophyte | Sexual | C | Wind | Zoochory | 774.6 |  |
| *Quercus robur* L. | Forest species | LC | Macrophanerophyte | Sexual | C | Wind | Zoochory | – |  |
| *Quercus rubra* L. | Forest species | LC | Macrophanerophyte | Sexual | C | Wind | Zoochory | 2694.0 |  |
| *Ranunculus auricomus* L. | Forest species | LC | – | – | CSR | Insects | Anemochory | – |  |
| *Ranunculus ficaria* L. | Forest species | LC | Geophyte | Mixed | CSR | Insects | Hemerochory | 1.0 |  |
| *Ribes rubrum* L. | Forest species | LC | Nanophanerophyte | Sexual | C | Insects | Zoochory | 9.2 |  |
| *Ribes uva-crispa* L. | Non-forest species | LC | Nanophanerophyte | Mixed | C | Insects | Zoochory | – |  |
| *Rosa* sp. | Forest species | LC | Nanophanerophyte | – | – | Insects | Zoochory | – |  |
| *Rubus* sp. | Forest species | LC | – | – | – | Insects | Zoochory | – |  |
| *Rumex* sp. | – | LC | Hemicryptophyte | – | – | Wind | Anemochory | – |  |
| *Sambucus nigra* L. | Forest species | LC | Nanophanerophyte | Sexual | C | Insects | Zoochory | 3.2 |  |
| *Solidago canadensis* aggr. | Non-forest species | LC | Hemicryptophyte | Mixed | C | Insects | Anemochory | 0.1 |  |
| *Sorbus x Sorbopyrus* | – | LC | – | – | – | Insects | – | – |  |
| *Stachys sylvatica* L. | Forest species | LC | Hemicryptophyte | Mixed | CS | Insects | Zoochory | 1.6 |  |
| *Stellaria media* aggr. | Non-forest species | LC | Therophyte | Sexual | CR | Insects | Anemochory | 0.5 |  |
| *Tanacetum* sp. | Non-forest species | LC | – | – | – | Insects | – | – |  |
| *Taraxacum officinale* aggr. | Non-forest species | LC | Hemicryptophyte | Sexual | CSR | Insects | Anemochory | 0.7 |  |
| *Taxus baccata* L. | Forest species | LC | Macrophanerophyte | Sexual | C | Wind | Zoochory | 51.6 |  |
| *Tilia platyphyllos* Scop. | Forest species | LC | Macrophanerophyte | Sexual | C | Insects | Anemochory | 112.0 |  |
| *Trifolium repens* L. | Non-forest species | LC | Hemicryptophyte | Mixed | CSR | Insects | Zoochory | 0.6 |  |
| *Ulmus glabra* Huds. | Forest species | LC | Macrophanerophyte | Sexual | C | Wind | Anemochory | 12.5 |  |
| *Urtica dioica* L. | Forest species | LC | Hemicryptophyte | Mixed | C | Wind | Anemochory | 0.1 |  |
| *Veronica chamaedrys* L. | Non-forest species | LC | Chamaephyte | Mixed | CSR | Insects | Anemochory | 0.2 |  |
| *Veronica montana* L. | Forest species | LC | Hemicryptophyte | Mixed | CSR | Insects | Anemochory | 0.3 |  |
| *Veronica serpyllifolia* L. | Non-forest species | LC | Hemicryptophyte | Mixed | CSR | Insects | Hydrochory | 0.1 |  |
|  |  |  |  |  |  |  |  |  | |

|  |  |  |  |  |  |  |  |  | |
| --- | --- | --- | --- | --- | --- | --- | --- | --- | --- |
| **Species** | **Habitat specificity^1^** | **Red List^2^** | **Plant life form^3^** | **Reproduction type^3^** | **Ecological strategy^3, 4^** | **Pollination syndrome^3^** | **Seed dispersal type^5^** | **Mean seed mass (mg)^3^** | |
|  |  |  |  |  |  |  |  |  |  |
| *Viburnum lantana* L. | Forest species | LC | Nanophanerophyte | Sexual | CS | Insects | Zoochory | – |  |
| *Viburnum opulus* L. | Forest species | LC | Nanophanerophyte | Sexual | C | Insects | Zoochory | 25.5 |  |
| *Viburnum rhytidophyllum* Hemsl. | – | LC | – | – | – | Insects | Zoochory | – |  |
| *Vicia cracca* L. s.l. | Non-forest species | LC | Hemicryptophyte | Mixed | C | Insects | Autochory | 14.3 |  |
| *Vicia sepium* L. | Non-forest species | LC | Hemicryptophyte | Mixed | C | Insects | Autochory | 21.4 |  |
| *Viola reichenbachiana* Boreau | Forest species | LC | Hemicryptophyte | Mixed | CSR | Insects | Autochory | 4.0 |  |

^1^ Delarze, R. et al. 2015. Lebensräume der Schweiz, 3rd edn. – Ott Verlag.

^2^ Red list of vascular plants: Bornand, C. et al. 2016. Rote Liste Gefässpflanzen. Gefährdete Arten der Schweiz. – Bundesamt für Umwelt, Bern und Info Flora, Genf. Umwelt-Vollzug Nr. 1621. Threat categories are: LC = least concern, NT = near threatened, VU = vulnerable

^3^ Klotz, S. et al. 2002. BIOLFLOR – Eine Datenbank mit Biologisch-Ökologischen Merkmalen zur Flora von Deutschland. – LandWirtschaftsverlag, Bonn. <http://www2.ufz.de/biolflor> accessed 10 May 2017.

^4^ Ecological strategy following ‘Grime, J. P. 1979. Plant strategies and vegetation processes. – Wiley’: C = competitive, S = stress tolerant, R = ruderal

^5^ Müller-Schneider, P. 1986. Verbreitungsbiologie der Blütenpflanzen Graubündens 85. Heft. – Veröffentl Geobot Inst ETH, Stiftung Rübel.b) Ants

|  |  |  |  |  | |  |  |  |
| --- | --- | --- | --- | --- | --- | --- | --- | --- |
| **Species** | **Habitat specifity^1^** | **Red List^2^** | **Subfamily** | **Body size (mm)^3^** | | **Number of queens^4^** | **Main Food^5^** | **Main nest stratum^6^** |
|  |  |  |  |  | |  |  |  |
| *Aphaenogaster subterranea* (Latreille 1798) | Forest species | VU | Myrmicinae | 5 |  | – | Carbohydrates & animal matter | Soil & crevices |
| *Colobopsis truncata* (Spinola 1808) | Forest species |  | Formicinae | 4 |  | Monogynous | Carbohydrates & animal matter | Wood & litter |
| *Dolichoderus quadripunctatus* (Linnaeus 1771) | Forest species |  | Dolichoderinae | 4 |  | Monogynous | Carbohydrates | Wood & litter |
| *Formica cunicularia* Latreille 1798 | Open-land species |  | Formicinae | 7.5 |  | Monogynous | Carbohydrates & animal matter | Soil & crevices |
| *Formica fusca* Linnaeus 1758 | Generalist |  | Formicinae | 7 |  | Polygynous | Carbohydrates & animal matter | Soil & crevices |
| *Formica rufibarbis* Fabricius 1793 | Open-land species |  | Formicinae | 7.5 |  | Polygynous | Animal matter | Soil & crevices |
| *Lasius brunneus* (Latreille 1798) | Forest species |  | Formicinae | 4.5 |  | Monogynous | Carbohydrates | Wood & litter |
| *Lasius emarginatus* (Olivier 1792) | Open-land species |  | Formicinae | 4.5 |  | Monogynous | Carbohydrates & animal matter | Soil & crevices |
| *Lasius flavus* (Fabricius 1798) | Open-land species |  | Formicinae | 4.8 |  | Oligogynous | Carbohydrates | Soil & crevices |
| *Lasius fuliginosus* (Latreille 1798) | Forest species |  | Formicinae | 6 |  | Polygynous | Carbohydrates & animal matter | Wood & litter |
| *Lasius mixtus* (Nylander 1846) | Generalist |  | Formicinae | 4.5 |  | Oligogynous | – | Soil & crevices |
| *Lasius myops* Forel 1894 | Open-land species |  | Formicinae | 3.6 |  | – | Carbohydrates & animal matter | Soil & crevices |
| *Lasius niger* (Linnaeus 1758) | Open-land species |  | Formicinae | 5 |  | Monogynous | Carbohydrates & animal matter | Soil & crevices |
| *Lasius platythorax* Seifert 1991 | Forest species | * | Formicinae | 5 |  | Monogynous | Carbohydrates & animal matter | Both |
| *Lasius psammophilus* Seifert 1992 | Open-land species | * | Formicinae | 4 |  | – | Carbohydrates & animal matter | Soil & crevices |
| *Lasius sabularum* (Bondroit 1918) | Generalist | * | Formicinae | 4.4 |  | – | Carbohydrates | Wood & litter |
| *Myrmecina graminicola* (Latreille 1802) | Generalist |  | Myrmicinae | 3.7 |  | Oligogynous | Animal matter | Soil & crevices |
| *Myrmica rubra* (Linnaeus 1758) | Generalist |  | Myrmicinae | 6 |  | Polygynous | Carbohydrates & animal matter | Both |
|  |  |  |  |  | |  |  |  |
| **Species** | **Habitat specifity^1^** | **Red List^2^** | **Subfamily** | **Body size (mm)^3^** | | **Number of queens^4^** | **Main Food^5^** | **Main nest stratum** |
|  |  |  |  |  | |  |  |  |
| *Myrmica ruginodis* Nylander 1846 | Forest species |  | Myrmicinae | 6 |  | Polygynous | Carbohydrates & animal matter | Both |
| *Myrmica sabuleti* Meinert 1861 | Open-land species |  | Myrmicinae | 5 |  | Polygynous | Carbohydrates & animal matter | Soil & crevices |
| *Myrmica scabrinodis* Nylander 1846 | Open-land species |  | Myrmicinae | 6 |  | Polygynous | Carbohydrates & animal matter | Soil & crevices |
| *Myrmica schencki* Viereck 1903 | Open-land species |  | Myrmicinae | 5.5 |  | Polygynous | Animal matter | Soil & crevices |
| *Myrmica specioides* Bondroit 1918 | Open-land species | VU | Myrmicinae | 4.5 |  | Oligogynous | – | Soil & crevices |
| *Solenopsis fugax* (Latreille 1798) | Open-land species |  | Myrmicinae | 3 |  | Polygynous | Animal matter | Soil & crevices |
| *Stenamma debile* (Förster 1850) | Forest species |  | Myrmicinae | 4.3 |  | Monogynous | Animal matter | Wood & litter |
| *Stenamma striatulum* Emery 1895 | Forest species | NT | Myrmicinae | 3.4 |  | – | – | Wood & litter |
| *Temnothorax affinis* (Mayr 1855) | Forest species |  | Myrmicinae | 3.5 |  | Monogynous | Carbohydrates & animal matter | Wood & litter |
| *Tetramorium* cf. *caespitum* (Linnaeus 1758) | Open-land species |  | Myrmicinae | 4 |  | Monogynous | Grains^7^ | Soil & crevices |

^1^ Forest species here include all species that primarily nest in wood, even if they can also be found in open habitats with single trees present, e.g. orchards.

^2^ No recent red list for the ants of Switzerland exists. Red List information in this table thus follows ‘Agosti, D. and Cherix, D. 1994. Rote Liste der gefährdeten Ameisen der Schweiz. In: Duelli, P. (ed.) Rote Listen der gefährdeten Tierarten der Schweiz. – Bundesamt für Umwelt, Wald und Landschaft, Bern. pp. 45–47.’ Threat categories were adjusted in July 2009 to IUCN categories by the Bundesamt für Umwelt <www.bafu.ch> according to the guidelines published on <http://www.artenschutz.ch/rlist.htm#2> assessed 14 July 2017. Threat categories are: NT = near threatened, VU = vulnerable. Species marked with a (*) were not considered as species-level taxa or not yet distinguished from sibling species in the literature cited by Agosti and Cherix (1994) to identify Swiss ants. It is thus likely their status was not evaluated for the Red List, even though they were already described as separate species at the time of publication in papers by Bernhard Seifert published in 1988-1992. This affects *Lasius plathythorax*, *L. psammophilus* and *L. sabularum*. Many other species have changed names since the publication of the original Red List, but their synonymy is clear. *Tetramorium caespitum* as it was defined at the time of the publication of the red list in 1994 is now recognized to constitute several species, not all of which have already been named, and whose distribution in Switzerland is yet incompletely known. Species considered to be not threatened were not entered into the original list, thus there are none classified into a category equivalent to the IUCN category least concern (LC). All species not marked with an asterisk were part of the keys recommended by Agosti and Cherix (1994) and thus all such species not assigned to a threat category were likely considered LC by the authors of this list.

^3^ Maximum total length of workers, including major workers, in species where these forage.

^4^ We were interested in the maximum number of queens per colony, thus for a species that has colonies that are monogynous and colonies that are oligogynous the latter would be entered.

^5^ Carbohydrates include nectar and animal secretions like honeydew, animal matter includes carrion and prey.

^6^ Nest substrate categories were combined to avoid small sample sizes. This means that e.g. the category Wood & crevices includes species using either of these substrates or both.

^7^ The species also uses carbohydrate and animal matter food resources. However, the categorization reflects,,that it is more granivorous than other species in these communitiesc) Spiders

|  |  |  |  |  | |  | | |  |  |  |
| --- | --- | --- | --- | --- | --- | --- | --- | --- | --- | --- | --- |
| **Species** | **Family** | **Habitat specificity^1, 2^** | **Red List^3^** | **Mean body size (mm)^2^** | | **Hunting mode^4, 5^** | | |  |  |  |
|  |  |  |  |  | |  | | |  |  |  |
| *Agyneta rurestris* (C.L. Koch, 1836) | Linyphiidae | Generalist | * | 2.13 |  | | Web building | | |  |  |
| *Alopecosa pulverulenta* (Clerck, 1757) | Lycosidae | Open-land species | * | 7.00 |  | | Hunting | | |  |  |
| *Amaurobius ferox* (Walckenaer, 1830) | Amaurobiidae | Generalist | * | 9.00 |  | | Web building | | |  |  |
| *Anyphaena accentuata* (Walckenaer, 1802) | Anyphaenidae | Forest species | * | 5.50 |  | | Hunting | | |  |  |
| *Apostenus fuscus* Westring, 1851 | Liocranidae | Generalist | * | 3.07 |  | | Hunting | | |  |  |
| *Atypus piceus* (Sulzer, 1776) | Atypidae | Generalist | V | 9.09 |  | | Hunting | | |  |  |
| *Centromerus serratus* (O.P.-Cambridge, 1875) | Linyphiidae | Generalist | * | 1.70 |  | | Web building | | |  |  |
| *Centromerus sylvaticus* (Blackwall, 1841) | Linyphiidae | Generalist | * | 3.75 |  | | Web building | | |  |  |
| *Ceratinella brevis* (Wider, 1834) | Linyphiidae | Generalist | * | 1.80 |  | | Web building | | |  |  |
| *Ceratinella scabrosa* (O.P.-Cambridge, 1871) | Linyphiidae | Generalist | * | 1.90 |  | | Web building | | |  |  |
| *Cetonana laticeps* (Canestrini, 1868) | Trachelidae | – | * | 6.25 |  | | – | | |  |  |
| *Cicurina cicur* (Fabricius, 1793) | Dictynidae | Generalist | * | 6.00 |  | | Web building | | |  |  |
| *Clubiona comta* (C.L. Koch, 1839) | Clubionidae | Forest species | * | 4.29 |  | | Hunting | | |  |  |
| *Clubiona terrestris* Westring, 1851 | Clubionidae | Forest species | * | 6.54 |  | | Hunting | | |  |  |
| *Cnephalocotes obscurus* (Blackwall, 1834) | Linyphiidae | Open-land species | * | 1.70 |  | | Web building | | |  |  |
| *Coelotes terrestris* (Wider, 1834) | Agelenidae | Forest species | * | 9.59 |  | | Web building | | |  |  |
| *Dicymbium nigrum* (Blackwall, 1834) | Linyphiidae | Generalist | * | 2.14 |  | | Web building | | |  |  |
| *Diplocephalus latifrons* (O.P.-Cambridge, 1863) | Linyphiidae | Generalist | * | 1.71 |  | | Web building | | |  |  |
| *Diplocephalus picinus* (Blackwall, 1841) | Linyphiidae | Forest species | * | 1.74 |  | | Web building | | |  |  |
| *Diplostyla concolor* (Wider, 1834) | Linyphiidae | Generalist | * | 2.75 |  | | Web building | | |  |  |
| *Drassyllus praeficus* (L. Koch, 1866) | Gnaphosidae | Open-land species | V | 6.50 |  | | Hunting | | |  |  |
| *Drassyllus pusillus* (C.L. Koch, 1833) | Gnaphosidae | Open-land species | * | 4.75 |  | | Hunting | | |  |  |
|  |  |  |  |  |  | |  | | |  |  |
| **Species** | **Family** | **Habitat specificity^1, 2^** | **Red List^3^** | **Mean body size (mm)^2^** | | **Hunting mode^4, 5^** | | |  |  |  |
|  |  |  |  |  |  | |  | | |  |  |
| *Dysdera erythrina* (Walckenaer, 1802) | Dysderidae | Generalist | * | 8.88 |  | | Hunting | | |  |  |
| *Enoplognatha latimana* Hippa & Oksala, 1982 | Theridiidae | Open-land species | * | 4.67 |  | | Web building | | |  |  |
| *Enoplognatha ovata* (Clerck, 1757) | Theridiidae | Generalist | * | 4.87 |  | | Web building | | |  |  |
| *Enoplognatha thoracica* (Hahn, 1833) | Theridiidae | Generalist | * | 3.78 |  | | Web building | | |  |  |
| *Entelecara acuminata* (Wider, 1834) | Linyphiidae | Generalist | * | 2.20 |  | | Web building | | |  |  |
| *Episinus truncatus* Latreille, 1809 | Theridiidae | Generalist | * | 5.30 |  | | Web building | | |  |  |
| *Erigone atra* Blackwall, 1833 | Linyphiidae | Generalist | * | 2.20 |  | | Web building | | |  |  |
| *Erigone dentipalpis* (Wider, 1834) | Linyphiidae | Generalist | * | 2.33 |  | | Web building | | |  |  |
| *Ero furcata* (Villers, 1789) | Mimetidae | Generalist | * | 3.95 |  | | Hunting | | |  |  |
| *Gonatium rubellum* (Blackwall, 1841) | Linyphiidae | Forest species | * | 3.20 |  | | Web building | | |  |  |
| *Gongylidium rufipes* (Linnaeus, 1758) | Linyphiidae | Generalist | * | 2.75 |  | | Web building | | |  |  |
| *Hahnia helveola* Simon, 1875 | Hahniidae | Forest species | * | 2.45 |  | | Web building | | |  |  |
| *Hahnia nava* (Blackwall, 1841) | Hahniidae | Open-land species | * | 1.75 |  | | Web building | | |  |  |
| *Hahnia pusilla* C.L. Koch, 1841 | Hahniidae | Generalist | * | 1.40 |  | | Web building | | |  |  |
| *Haplodrassus silvestris* (Blackwall, 1833) | Gnaphosidae | Forest species | * | 7.53 |  | | Hunting | | |  |  |
| *Harpactea hombergi* (Scopoli, 1763) | Dysderidae | Generalist | * | 5.00 |  | | Hunting | | |  |  |
| *Harpactea lepida* (C.L. Koch, 1838) | Dysderidae | Forest species | * | 6.00 |  | | Hunting | | |  |  |
| *Histopona torpida* (C.L. Koch, 1837) | Agelenidae | Forest species | * | 5.67 |  | | Web building | | |  |  |
| *Inermocoelotes inermis* (L. Koch, 1855) | Agelenidae | Forest species | * | 9.64 |  | | Web building | | |  |  |
| *Lathys humilis* (Blackwall, 1855) | Dictynidae | Generalist | * | 2.13 |  | | Web building | | |  |  |
| *Linyphia hortensis* Sundevall, 1830 | Linyphiidae | Forest species | * | 4.72 |  | | Web building | | |  |  |
| *Linyphia triangularis* (Clerck, 1757) | Linyphiidae | Generalist | * | 6.00 |  | | Web building | | |  |  |
| *Macrargus rufus* (Wider, 1834) | Linyphiidae | Forest species | * | 4.50 |  | | Web building | | |  |  |
|  |  |  |  |  |  | |  | | |  |  |
| **Species** | **Family** | **Habitat specificity^1, 2^** | **Red List^3^** | **Mean body size (mm)^2^** | | **Hunting mode^4, 5^** | | |  |  |  |
|  |  |  |  |  |  |  | | |  |  |  |
| *Maso sundevalli* (Westring, 1851) | Linyphiidae | Generalist | * | 1.52 |  | Web building | | |  |  |  |
| *Mermessus trilobatus* (Emerton, 1882) | Linyphiidae | Generalist | – | 1.85 |  | Web building | | |  |  |  |
| *Metellina merianae* (Scopoli, 1763) | Tetragnathidae | Generalist | * | 9.65 |  | Web building | | |  |  |  |
| *Metellina segmentata* (Clerck, 1757) | Tetragnathidae | Generalist | * | 7.25 |  | Web building | | |  |  |  |
| *Micaria pulicaria* (Sundevall, 1831) | Gnaphosidae | Open-land species | * | 3.50 |  | Hunting | | |  |  |  |
| *Micrargus herbigradus* (Blackwall, 1854) | Linyphiidae | Generalist | * | 2.08 |  | Web building | | |  |  |  |
| *Micrargus subaequalis* (Westring, 1851) | Linyphiidae | Open-land species | * | 1.80 |  | Web building | | |  |  |  |
| *Microneta viaria* (Blackwall, 1841) | Linyphiidae | Forest species | * | 2.25 |  | Web building | | |  |  |  |
| *Monocephalus fuscipes* (Blackwall, 1836) | Linyphiidae | Forest species | * | 2.21 |  | Web building | | |  |  |  |
| *Neottiura bimaculata* (Linnaeus, 1767) | Theridiidae | Generalist | * | 2.60 |  | Web building | | |  |  |  |
| *Neriene clathrata* (Sundevall, 1830) | Linyphiidae | Generalist | * | 4.21 |  | Web building | | |  |  |  |
| *Neriene emphana* (Walckenaer, 1841) | Linyphiidae | Forest species | * | 3.80 |  | Web building | | |  |  |  |
| *Neriene peltata* (Wider, 1834) | Linyphiidae | Forest species | * | 5.25 |  | Web building | | |  |  |  |
| *Oedothorax apicatus* (Blackwall, 1850) | Linyphiidae | Open-land species | * | 2.89 |  | Web building | | |  |  |  |
| *Oedothorax fuscus* (Blackwall, 1834) | Linyphiidae | Open-land species | * | 2.15 |  | Web building | | |  |  |  |
| *Ozyptila praticola* (C.L. Koch, 1837) | Thomisidae | Generalist | * | 2.91 |  | Hunting | | |  |  |  |
| *Ozyptila simplex* (O.P.-Cambridge, 1862) | Thomisidae | Open-land species | * | 4.55 |  | Hunting | | |  |  |  |
| *Pachygnatha degeeri* Sundevall, 1830 | Tetragnathidae | Open-land species | * | 3.56 |  | Hunting | | |  |  |  |
| *Paidiscura pallens* (Blackwall, 1834) | Theridiidae | Forest species | * | 1.85 |  | Web building | | |  |  |  |
| *Palliduphantes pallidus* (O.P.-Cambridge, 1871) | Linyphiidae | Forest species | * | 1.95 |  | Web building | | |  |  |  |
| *Panamomops mengei* Simon, 1926 | Linyphiidae | Forest species | D | 1.50 |  | Web building | | |  |  |  |
| *Parasteatoda simulans* (Thorell, 1875) | Theridiidae | Forest species | * | 4.20 |  | Web building | | |  |  |  |
| *Parasteatoda tepidariorum* (C.L. Koch, 1841) | Theridiidae | Generalist | * | 5.50 |  | Web building | | |  |  |  |
|  |  |  |  |  |  |  | | |  |  |  |
| **Species** | **Family** | **Habitat specificity^1, 2^** | **Red List^3^** | **Mean body size (mm)^2^** | | **Hunting mode^4, 5^** | | |  |  |  |
|  |  |  |  |  |  |  | | |  |  |  |
| *Pardosa amentata* (Clerck, 1757) | Lycosidae | Generalist | * | 6.00 |  | Hunting | | |  |  |  |
| *Pardosa hortensis* (Thorell, 1872) | Lycosidae | Generalist | * | 4.65 |  | Hunting | | |  |  |  |
| *Pardosa pullata* (Clerck, 1757) | Lycosidae | Open-land species | * | 4.50 |  | Hunting | | |  |  |  |
| *Pardosa saltans* Töpfer-Hofmann, 2000 | Lycosidae | Forest species | * | 5.51 |  | Hunting | | |  |  |  |
| *Philodromus albidus* Kulczyński, 1911 | Philodromidae | Forest species | * | 3.48 |  | Hunting | | |  |  |  |
| *Philodromus aureolus* (Clerck, 1757) | Philodromidae | Generalist | * | 5.00 |  | Hunting | | |  |  |  |
| *Pholcomma gibbum* (Westring, 1851) | Theridiidae | Generalist | * | 1.66 |  | Web building | | |  |  |  |
| *Phrurolithus festivus* (C.L. Koch, 1835) | Phrurolithidae | Generalist | * | 2.68 |  | Hunting | | |  |  |  |
| *Piratula hygrophila* (Thorell, 1872) | Lycosidae | Generalist | * | 5.31 |  | Hunting | | |  |  |  |
| *Piratula latitans* (Blackwall, 1841) | Lycosidae | Open-land species | * | 3.50 |  | Hunting | | |  |  |  |
| *Piratula uliginosa* (Thorell, 1856) | Lycosidae | Open-land species | * | 4.50 |  | Hunting | | |  |  |  |
| *Pocadicnemis pumila* (Blackwall, 1841) | Linyphiidae | Generalist | * | 1.90 |  | Web building | | |  |  |  |
| *Porrhomma microphthalmum* (O.P.-Cambridge, 1871) | Linyphiidae | Open-land species | * | 1.85 |  | Web building | | |  |  |  |
| *Saaristoa abnormis* (Blackwall, 1841) | Linyphiidae | Generalist | * | 3.41 |  | Web building | | |  |  |  |
| *Scotina celans* (Blackwall, 1841) | Liocranidae | Generalist | V | 3.09 |  | Hunting | | |  |  |  |
| *Tapinocyba insecta* (L. Koch, 1869) | Linyphiidae | Forest species | * | 1.65 |  | Web building | | |  |  |  |
| *Tegenaria silvestris* L. Koch, 1872 | Agelenidae | Generalist | * | 5.50 |  | Web building | | |  |  |  |
| *Tenuiphantes flavipes* (Blackwall, 1854) | Linyphiidae | Forest species | * | 2.15 |  | Web building | | |  |  |  |
| *Tenuiphantes tenuis* (Blackwall, 1852) | Linyphiidae | Generalist | * | 2.94 |  | Web building | | |  |  |  |
| *Tenuiphantes zimmermanni* (Bertkau, 1890) | Linyphiidae | Generalist | * | 2.56 |  | Web building | | |  |  |  |
| *Tetragnatha nigrita* Lendl, 1886 | Tetragnathidae | Generalist | * | 7.00 |  | Web building | | |  |  |  |
| *Tetragnatha obtusa* C.L. Koch, 1837 | Tetragnathidae | Generalist | * | 6.10 |  | Web building | | |  |  |  |
| *Textrix denticulata* (Olivier, 1789) | Agelenidae | Generalist | * | 7.43 |  | Web building | | |  |  |  |
|  |  |  |  |  |  |  | | | | | |
| **Species** | **Family** | **Habitat specificity^1, 2^** | **Red List^3^** | **Mean body size (mm)^2^** | | **Hunting mode^4, 5^** | | | | | |
|  |  |  |  |  |  | | |  | | |  |
| *Theridion pinastri* L. Koch, 1872 | Theridiidae | Generalist | * | 3.50 |  | | | Web building | | |  |
| *Tiso vagans* (Blackwall, 1834) | Linyphiidae | Generalist | * | 2.03 |  | | | Web building | | |  |
| *Trachyzelotes pedestris* (C.L. Koch, 1837) | Gnaphosidae | Open-land species | * | 6.50 |  | | | Hunting | | |  |
| *Trochosa ruricola* (De Geer, 1778) | Lycosidae | Open-land species | * | 8.94 |  | | | Hunting | | |  |
| *Trochosa terricola* Thorell, 1856 | Lycosidae | Generalist | * | 8.42 |  | | | Hunting | | |  |
| *Walckenaeria acuminata* Blackwall, 1833 | Linyphiidae | Generalist | * | 3.75 |  | | | Web building | | |  |
| *Walckenaeria alticeps* (Denis, 1952) | Linyphiidae | Forest species | V | 2.35 |  | | | Web building | | |  |
| *Walckenaeria atrotibialis* (O.P.-Cambridge, 1878) | Linyphiidae | Generalist | * | 2.30 |  | | | Web building | | |  |
| *Walckenaeria corniculans* (O.P.-Cambridge, 1875) | Linyphiidae | Forest species | * | 2.68 |  | | | Web building | | |  |
| *Walckenaeria cucullata* (C.L. Koch, 1836) | Linyphiidae | Forest species | * | 2.10 |  | | | Web building | | |  |
| *Walckenaeria dysderoides* (Wider, 1834) | Linyphiidae | Generalist | * | 1.95 |  | | | Web building | | |  |
| *Walckenaeria incisa* (O.P.-Cambridge, 1871) | Linyphiidae | Forest species | D | 2.45 |  | | | Web building | | |  |
| *Walckenaeria nudipalpis* (Westring, 1851) | Linyphiidae | Generalist | * | 3.20 |  | | | Web building | | |  |
| *Walckenaeria vigilax* (Blackwall, 1853) | Linyphiidae | Open-land species | * | 2.10 |  | | | Web building | | |  |
| *Zelotes apricorum* (L. Koch, 1876) | Gnaphosidae | Generalist | * | 6.84 |  | | | Hunting | | |  |
| *Zilla diodia* (Walckenaer, 1802) | Araneidae | Forest species | * | 4.30 |  | | | Web building | | |  |
| *Zodarion italicum* (Canestrini, 1868) | Zodariidae | Open-land species | * | 2.58 |  | | | Hunting | | |  |
| *Zodarion rubidum* Simon, 1914 | Zodariidae | Open-land species | * | 3.60 |  | | | Hunting | | |  |

^1^ Hänggi, A. et al. 1995. Lebensräume Mitteleuropäischer Spinnen. – Miscellanea Faunistica Helvetiae 4, Centre de cartographie de la faune (CSCF).

^2^ Nentwig, W. et al. 2017. Spiders of Europe. – <www.araneae.unibe.ch> version 07.2017.

^3^ There does not exist a Red List of Switzerland. Therefore, the Red List of Baden-Württemberg is shown instead: Nährig, D. and Harms, K. H. 2003. Rote Liste und Checkliste der Spinnentiere (Arachnida) Baden-Württembergs. – Naturschutz-Praxis, Artenschutz. Threat categories are: * = least concern, D = data deficient, V = near threatened

^4^ Wiki der Arachnologischen Gesellschaft e.V., ‘Hauptseite’, <https://wiki.arages.de/index.php?title=Hauptseite&oldid=91730> accessed 23 May 2017.

^5^ Hunting including active hunting and ambushing

^6^ Bell, J. R. et al. 2005. Ballooning dispersal using silk: world fauna, phylogenies, genetics and models. – B. Entomol. Res. 95: 69–114.
