# Supplementary material for: Diverse effects of degree of urbanisation and forest size on species richness and functional diversity of plants, and ground surface-active ants and spiders
Source: PLoS One. 2018 Jun 19;13(6):e0199245. doi: 10.1371/journal.pone.0199245 (PMC6007905; doi:10.1371/journal.pone.0199245)
Supplement: S4 Table — Results of Pearson’s (r) and Spearman’s rank (rs) correlation, Contingency table (χ2-test) and Kruskal-Wallis test examining the relationship between observed species richness and rarefied species richness (a) and among landscape and forest characteristics for all three taxonomic groups (b), in the vegetation plots (c) and in the trap-grid system (d) (DOCX) [file pone.0199245.s005.docx]

**S4 Table. Correlations within and among landscape and site characteristics.** Results of Pearson’s (r) and Spearman’s rank (r_s_) correlation, Contingency table (χ^2^-test) and Kruskal-Wallis test examining the relationship between observed species richness and rarefied species richness (a) and among landscape and forest characteristics for all three taxonomic groups (b), in the vegetation plots (c) and in the trap-grid system (d).

a) Species richness and functional richness

| **Observed species richness vs. sample-based rarefied species richness** | | | |
| --- | --- | --- | --- |
|  | Ants | Pearson | **r = 1.00, n = 26, P < 0.001** |
|  | Spiders | Pearson | **r = 1.00, n = 26, P < 0.001** |
|  | |  |  |

b) Variables considered in all three taxonomic groups

| **Landscape characteristics**  Percentage cover of … | |  |  |
| --- | --- | --- | --- |
| Radius = 200 m |  |  |  |
| Sealed area vs. forest cover | | Spearman | **r_s_ = –0.47, n = 26, P = 0.015** |
| Sealed area vs. agricultural land | | Spearman | **r_s_ = –0.54, n = 26, P = 0.005** |
| Sealed area vs. urban green space | | Spearman | **r_s_ = 0.65, n = 26, P < 0.001** |
|  | |  |  |
| Radius = 500 m |  |  |  |
| Sealed area vs. forest cover | | Spearman | **r_s_ = –0.66, n = 26, P < 0.001** |
| Sealed area vs. agricultural land | | Spearman | **r_s_ =–0.57, n = 26, P = 0.002** |
| Sealed area vs. urban green space | | Spearman | **r_s_ =0.52, n = 26, P = 0.007** |
|  |  |  |  |
| Sealed area: R = 200 m vs. R = 500 m | | Pearson | **r = 0.88, n = 26, P < 0.001** |

| **Shape index** |  |  |
| --- | --- | --- |
| Degree of urbanisation vs. shape index | χ^2^-test | χ^2^ = 3.87, df = 4, P = 0.42 |
| Forest size vs. shape index | χ^2^-test | χ^2^ = 8.06, df = 4, P = 0.089 |
|  |  |  |
| **Historical development** |  |  |
| Degree of urbanisation vs. history | χ^2^-test | χ^2^ = 6.09, df = 4, P = 0.19 |
| Forest size vs. history | χ^2^-test | **χ^2^ = 12.97, df = 4, P = 0.011** |
|  |  |  |
| **Recreational pressure** |  |  |
|  |  |  |
| Path density vs. total trampled area | Spearman | **r_s_ = 0.52, n = 26, P = 0.007** |

c) Variables considered in vegetation plots

| **Species richness vs. cover of ground vegetation** | Spearman | r_s_ = 0.19, n = 26, P = 0.36 | |
| --- | --- | --- | --- |
|  |  |  |  |
| **Soil and litter characteristics** |  |  | |
| Soil organic matter content vs.  Soil organic nitrogen content | Spearman | **r_s_ = 0.85, n = 26, P < 0.001** | |

d) Variables considered in trap-grid system

| **Soil and litter characteristics** | | |
| --- | --- | --- |
|  |  |  |
| Soil moisture vs. ln(soil organic matter content) | Pearson | **r = 0.40, n = 26, P = 0.044** |
| Soil pH vs. ln(soil organic matter content) | Spearman | **r_s_ = 0.57, n = 26, P = 0.002** |
| Soil moisture vs. litter moisture | Pearson | **r = 0.52, n = 26, P = 0.006** |
| Soil pH vs. litter pH | Spearman | **r_s_ = 0.56, n = 26, P = 0.003** |
