# Supplementary material for: Diverse effects of degree of urbanisation and forest size on species richness and functional diversity of plants, and ground surface-active ants and spiders
Source: PLoS One. 2018 Jun 19;13(6):e0199245. doi: 10.1371/journal.pone.0199245 (PMC6007905; doi:10.1371/journal.pone.0199245)
Supplement: S8 Table — Summary of ANCOVAs examining the effects of degree of urbanisation, forest size and shape, forest management (time since last thinning), disturbance (indicated by path density), canopy closure, soil characteristics (moisture, pH, soil organic nitrogen (orgN) and phosphorus (orgP) content) and cover of ground vegetation on functional dispersion of vascular plants. (DOCX) [file pone.0199245.s009.docx]

**S8 Table.** **Functional dispersion: Summary of ANCOVAs of plants.** Summary of ANCOVAs examining the effects of degree of urbanisation, forest size and shape, forest management (time since last thinning), disturbance (indicated by path density), canopy closure, soil characteristics (moisture, pH, soil organic nitrogen (orgN) and phosphorus (orgP) content) and cover of ground vegetation on functional dispersion of vascular plants.

|  | |  | Functional dispersion | | |  |
| --- | --- | --- | --- | --- | --- | --- |
|  | |  | df | F | P |  |
|  | |  |  |  |  |  |
|  | Degree of urbanisation |  | 2,16 | 3.92 | **0.041** |  |
|  | Forest size |  | 2,16 | 3.68 | **0.049** |  |
|  | Shape index |  | 2,16 | 1.67 | 0.34 |  |
|  | Time since last thinning |  | 2,16 | 4.94 | **0.021** |  |
|  | Path density |  | – | – | – |  |
|  | Canopy closure |  | – | – | – |  |
|  | Soil moisture content |  | – | – | – |  |
|  | Soil pH |  | – | – | – |  |
|  | Soil organic nitrogen content^1^ |  | – | – | – |  |
|  | Soil organic phosphorus content^1^ |  | 1,16 | 1.56 | 0.23 |  |
|  | Cover of ground vegetation |  | – | – | – |  |
|  | Degree of urbanisation*forest size |  | – | – | – |  |

Significant P-values (< 0.05) are in bold

^1^ log-transformed

– Factor was excluded from the model by step-wise reduction
