# Supplementary material for: Diverse effects of degree of urbanisation and forest size on species richness and functional diversity of plants, and ground surface-active ants and spiders
Source: PLoS One. 2018 Jun 19;13(6):e0199245. doi: 10.1371/journal.pone.0199245 (PMC6007905; doi:10.1371/journal.pone.0199245)
Supplement: S9 Table — Summary of ANCOVAs examining the effects of degree of urbanisation, forest size and shape, forest management (time since last thinning), disturbance (indicated by path density), canopy closure, soil organic matter content, litter characteristics (moisture, pH) and structural diversity measures (litter biomass, vegetation structure and amount of dead wood) on functional dispersion of ants and spiders. (DOCX) [file pone.0199245.s010.docx]

**S9 Table. Functional dispersion: Summary of ANCOVAs of ants and spiders.**  Summary of ANCOVAs examining the effects of degree of urbanisation, forest size and shape, forest management (time since last thinning), disturbance (indicated by path density), canopy closure, soil organic matter content, litter characteristics (moisture, pH) and structural diversity measures (litter biomass, vegetation structure and amount of dead wood) on functional dispersion of ants and spiders.

|  |  | Functional dispersion | | | |
| --- | --- | --- | --- | --- | --- |
|  |  |  | df | F | P |
| **Ants** | |  |  |  |  |
|  | Degree of urbanisation |  | 2,13 | 1.82 | 0.20 |
|  | Forest size |  | 2,13 | 3.68 | 0.054 |
|  | Shape index |  | – | – | – |
|  | Time since last thinning |  | – | – | – |
|  | Path density |  | – | – | – |
|  | Canopy closure |  | 1,13 | 3.19 | 0.097 |
|  | Soil organic matter content^1^ |  | 1,13 | 0.18 | 0.68 |
|  | Litter moisture content |  | 1,13 | 12.63 | **0.004** |
|  | Litter pH |  | 1,13 | 3.29 | 0.093 |
|  | Amount of litter biomass^1^ |  | – | – | – |
|  | Vegetation structure^1^ |  | – | – | – |
|  | Amount of dead wood |  | – | – | – |
|  | Degree of urbanisation*forest size |  | 4,13 | 2.76 | 0.074 |
|  |  |  |  |  |  |
| **Spiders** | |  |  |  |  |
|  | Degree of urbanisation |  | 2,20 | 1.91 | 0.17 |
|  | Forest size |  | 2,20 | 2.86 | 0.081 |
|  | Shape index |  | – | – | – |
|  | Time since last thinning |  | – | – | – |
|  | Path density |  | – | – | – |
|  | Canopy closure |  | – | – | – |
|  | Soil organic matter content^1^ |  | – | – | – |
|  | Litter moisture content |  | – | – | – |
|  | Litter pH |  | – | – | – |
|  | Amount of litter biomass^1^ |  | – | – | – |
|  | Vegetation structure^1^ |  | 1,20 | 1.33 | 0.26 |
|  | Amount of dead wood |  | – | – | – |
|  | Degree of urbanisation*forest size |  | – | – | – |

Significant P-values (<0.05) are in bold

^1^ log-transformed

– Factor was excluded from the model by step-wise reduction
